# Gut Microbiota and Feeding Patterns of the Antarctic Fairy Shrimp (*Branchinecta gaini* Daday, 1910): A Metabarcoding Perspective

**DOI:** 10.1007/s00248-026-02820-4

**Published:** 2026-07-02

**Authors:** Stanisław Cukier, Jan Gawor, Jakub Grzesiak

**Affiliations:** 1 Institute of Biochemistry and Biophysics, Polish Academy of Sciences, Pawińskiego 5A, 02-106 Warsaw, Poland; 2 Doctoral School of Molecular Biology and Biological Chemistry, Pawińskiego 5A, 02-106 Warsaw, Poland

**Keywords:** Gut-associated bacteria, Environmentally acquired microbiota, Freshwater zooplankton, Polar limnology, Microbial community assembly

## Abstract

Understanding how feeding ecology and environmental conditions shape gut microbiota is essential for interpreting host–microbe interactions in extreme environments. Here, we investigated the diet and gut-associated bacterial communities of the Antarctic fairy shrimp *Branchinecta gaini* across multiple postglacial freshwater ponds on King George Island. We combined microscopic gut content analysis with 18 S and 16 S rRNA gene metabarcoding and distinguished between gut content and gut tract–associated bacterial fractions in pooled, pond-level samples to assess the relative roles of diet, host filtering, and environmental context in structuring gut-associated communities. Our results reveal pronounced dietary flexibility of *B. gaini*, with strong site-specific differences in consumed eukaryotic taxa reflecting local resource availability. This trophic variability was mirrored by highly variable gut-associated bacterial communities, characterized by low taxonomic overlap among ponds and the absence of a stable core microbiota at the pooled sample level. Although bacterial assemblages differed between gut contents and gut tract, consistent at the composite-sample scale, this pattern suggests limited evidence for strong host filtering. Gut-associated communities retained pond-specific signatures, indicating a dominant role of environmental sourcing. Environmental drivers influenced different aspects of gut microbiome organization: hydrological connectivity and associated conductivity gradients were linked to shifts in bacterial community composition, whereas water temperature showed a non-linear association with bacterial alpha diversity but not with overall community structure. Water pH showed no detectable effect on either metric. Together, these findings indicate that gut-associated bacterial communities of *B. gaini*, as captured by pooled samples, largely reflect environmentally acquired assemblages shaped by opportunistic feeding and local environmental filtering. This ecological flexibility may represent a key strategy enabling persistence of *B. gaini* across highly heterogeneous Antarctic freshwater ecosystems.

**Supplementary Information:**

The online version contains supplementary material available at https://doi.org/10.1007/s00248-026-02820-4.

## Introduction

The Antarctic fairy shrimp *Branchinecta gaini* is the largest freshwater invertebrate inhabiting Antarctic inland water bodies and the only member of the order *Anostraca* that has successfully colonized freshwater habitats of the maritime Antarctica and the Antarctic Peninsula following the retreat of the last glaciation [1–4]. It is a ubiquitous inhabitant of ponds and lakes in this region and represents one of the few metazoan consumers consistently present in Antarctic freshwater ecosystems. As such, *B. gaini* constitutes an important biological link between microbial primary producers, detrital material, and higher trophic levels where present [5, 6].

Antarctic freshwater ecosystems are characterized by extreme environmental conditions, including strong seasonality, frequent freezing, and pronounced spatial isolation. These habitats typically support low diversity of macro-organisms but harbour diverse and productive microbial communities [7, 8]. Primary production is often limited and depends largely on allochthonous inputs, such as glacial meltwater sediments, wind-deposited marine algae, and guano from seabirds and marine mammals. Despite these constraints, *B. gaini* has successfully established widespread populations across a range of highly dynamic freshwater environments, highlighting its ecological flexibility and broad trophic niche [3, 5, 9].

King George Island (South Shetland Islands, maritime Antarctica) provides a particularly suitable setting for studying the ecology of *B. gaini*. The island combines relatively mild climatic conditions with high environmental heterogeneity, supporting numerous freshwater ponds that differ in hydrology, physicochemical characteristics, and organic matter inputs. These conditions allow *B. gaini* to complete its rapid life cycle and exploit variable food resources, making King George Island a natural laboratory for investigating dietary strategies and gut-associated microbial communities in Antarctic freshwater invertebrates [10].

Adaptation to Antarctic conditions in *B. gaini* is facilitated by the production of resistant cysts, enabling survival through freezing and desiccation. However, this strategy requires substantial energetic investment within a short growth period of only 2–3 months [3, 9, 11]. Most anostracans are non-selective filter feeders that capture bacteria, algae, and detrital particles using specialized appendages and possess robust mandibles capable of mechanically disrupting cell walls [12, 13]. Microscopy-based studies have shown that the diet of *B. gaini* includes large quantities of microbial cells, green algae, fungal material, and fragments of invertebrates, including conspecifics [5]. Although these polymer-rich resources can be nutritionally valuable if enzymatically degraded, low environmental temperatures in Antarctic freshwater systems may reduce digestive efficiency and limit nutrient assimilation [9, 14].

Microorganisms associated with ingested material or transiently present within the digestive tract may play an important complementary role in nutrient acquisition, either by increasing nutrient availability through extracellular enzymatic activity or by contributing directly as a food source for filter-feeding crustaceans [15].

At the same time, many crustaceans are considered to lack stable gut microbiomes due to the presence of the peritrophic matrix—a chitin-based structure lining the midgut that physically separates microbes from the gut epithelium and is continuously shed, thereby limiting long-term microbial colonization [16].

Nevertheless, studies on other anostracan species indicate that microbial attachment and biofilm formation on gut-associated structures can occur, suggesting that transient microbial associations may arise under certain environmental conditions [15, 17]. Whether such associations represent stable colonization or primarily reflect environmentally acquired, short-lived assemblages remains unresolved, particularly in polar freshwater systems.

Despite the ecological importance of *B. gaini*, its gut-associated microbial communities and their relationship to dietary strategies remain poorly understood. Previous research has focused mainly on the physical and chemical characteristics of Antarctic fairy shrimp habitats, while biological interactions within these ecosystems have received comparatively little attention [18–20]. To date, only a single study has examined the dietary composition of *B. gaini* using microscopy-based approaches [5], which are inherently limited to the detection of morphologically recognizable and undigested components. Furthermore, the only available study addressing the gut microbiota of *B. gaini* did not explicitly consider differences between gut-associated microhabitats, leaving unresolved the extent to which microbial assemblages reflect transient dietary inputs versus host-related filtering processes [21]. Here, we investigate how the diet and gut-associated microbial communities of *B. gaini* vary across Antarctic freshwater habitats. Specifically, we aim to (i) characterize the dietary composition of wild *B. gaini* populations across multiple ponds using complementary microscopy-based and amplicon sequencing approaches, (ii) assess whether bacterial communities associated with the gut tract, including structures influenced by the peritrophic matrix, primarily reflect transient environmental inputs or exhibit evidence of host-specific filtering, and (iii) evaluate the extent to which environmental heterogeneity among ponds shapes the composition of eukaryotic and bacterial components within the gut of *B. gaini*. Based on previous studies [5, 10, 21] indicating the ecological flexibility of *B*. *gaini* we hypothesize that diet composition differs substantially among ponds in response to local resource availability, that gut tract–associated bacterial communities are dominated by environmentally derived and highly variable taxa rather than a stable core microbiome, and that environmental conditions act as strong filters shaping both dietary intake and gut-associated bacterial community composition. By integrating dietary, microbiological, and environmental data, this study provides new insights into the ecological strategies enabling *B. gaini* to persist in the extreme and dynamic freshwater ecosystems of Antarctica.

## Materials and Methods

### Study Sites

**Fig. 1:**
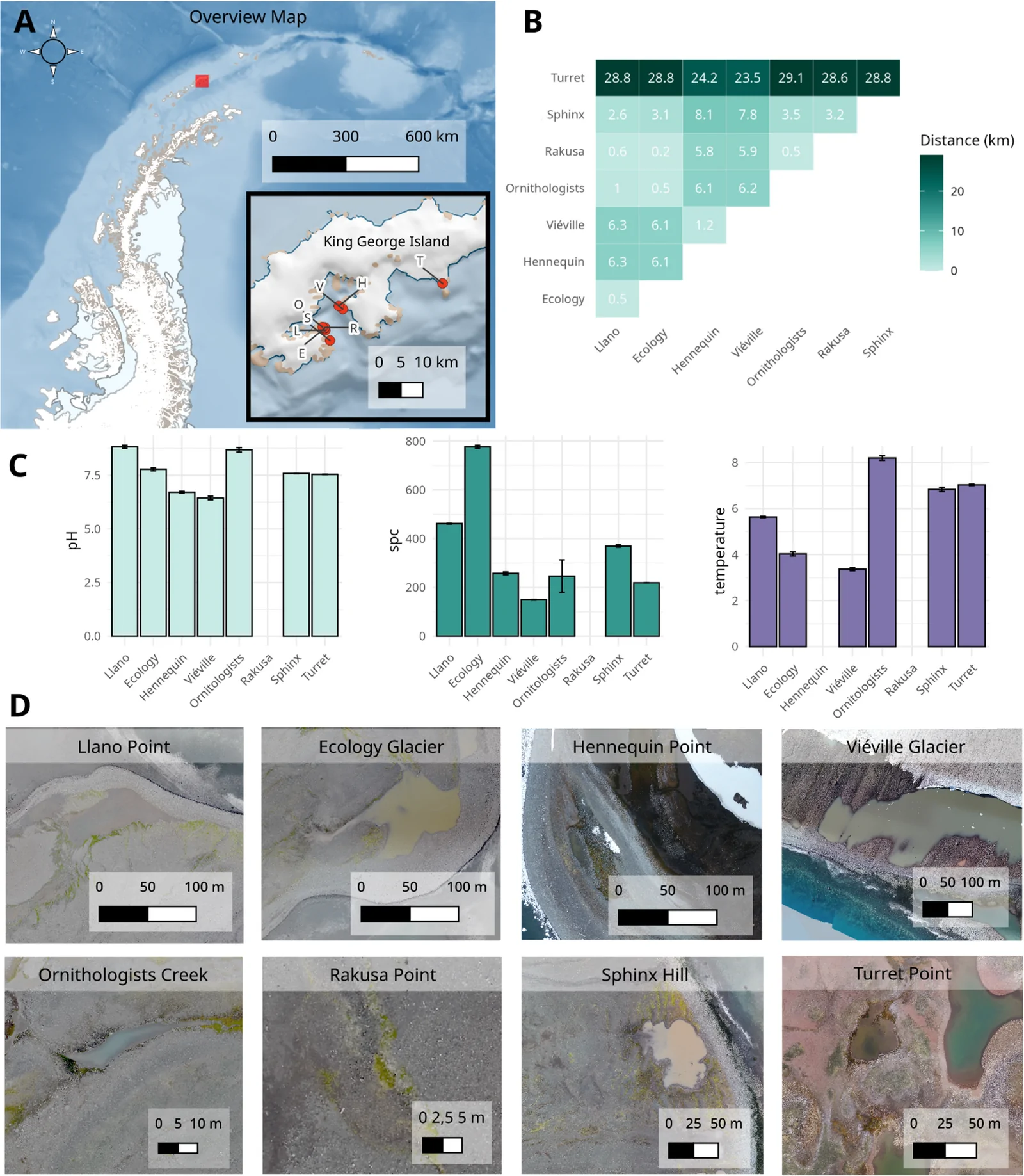
Overview of the sampling sites and environmental context. (**A**) Geographic distribution of the eight studied water bodies on King George Island, generated using the Quantarctica package (Norwegian Polar Institute). (**B**) Heatmap of pairwise geographic distances (km) between sampling sites, calculated from GNSS coordinates. (**C**) Physicochemical characteristics of the sampled waters at each site, including pH, temperature (°C), and electrical conductivity (µS cm⁻¹). Bars represent mean values, with error bars indicating standard deviations.(**D**) Orthophotos of individual sampling sites acquired using an unmanned aerial vehicle (UAV). Precise sampling locations are indicated based on GNSS coordinates;

The study was conducted in eight separate freshwater ponds located along the shores of Admiralty Bay and King George Bay (King George Island, South Shetland Islands, Maritime Antarctica) (Fig. 1A; Table 1). A series of hydrologically interconnected water bodies were treated as a single sampling site. During sampling, geographic coordinates were recorded using a GNSS receiver (GPSMAP^®^ 78, Garmin Ltd.), which enabled subsequent spatial analyses. Distances between localities were calculated using the *geosphere* package in the R programming environment [24]. Sampling sites were separated by distances ranging from 0.2 to 29.1 km (Fig. 1B).

**Table 1:** Description of sampling points

	Name (abbrev.)	Time of Sampling	Coordinates	Description
54	Llano Point (Llano)	12.12.2021	62°10’21” S 58°27’32” W	A shallow pond located along the shore of Suszczewski Cove, adjacent to the Ecology Glacier. The surrounding hills feature peat mounds approximately 100 cm thick, covered with Antarctic hair grass (*Deschampsia antarctica*) and mosses such as *Polytrichum alpinum* and *Drepanocladus uncinatus* [22]. The pond bottom consists of mixed silt and mud, with visible accumulations of dead organic matter. The water is slightly turbid. The pond likely formed within the past 30 years due to the retreat of the Ecology Glacier [23].
59	Ecology Glacier (Ecology)	17.12.2021	62°10’07” S 58°27’44” W	A complex of several interconnected ponds. The bottom is a mix of stony and muddy substrates. The water is turbid and greenish, indicating the presence of planktonic algae. Filamentous algae mats are present along the shores, where many *B*. *gaini* individuals become entangled and immobilized, leading to mortality. Nearby, the activity of birds such as skuas (*Stercorarius antarcticus*, *S*. *maccormicki*), Antarctic terns (*Sterna vittata*), and Antarctic fur seals (*Arctocephalus gazella*) can be observed. The immediate area is mostly devoid of vascular plants, but lichens and small patches of *Colobanthus quitensis* are present. The ponds likely formed within the past 30 years due to glacial retreat. Historical data suggest these ponds were initially connected to seawater, implying that *B*. *gaini* may have colonized them within the last few decades.
61	Hennequin Point (Hennequin)	21.12.2021	62°07’24” S 58°23’50” W	This site is located on the opposite side of Admiralty Bay from the Henryk Arctowski Polish Antarctic Station, near the Uruguayan summer station. It is an ephemeral, shallow puddle with a rocky bottom. Microbial mats are visible between the rocks, and the site is surrounded by dense moss patches. The area serves as a nesting site for south polar skuas (*Stercorarius maccormicki*).
63	Viéville Glacier (Viéville)	24.12.2021	62°07’48” S 58°22’47” W	A relatively large glacial lake situated at the base of the Viéville Glacier. The site likely formed in recent decades as the glacier front has retreated from the shoreline. The water is moderately turbid, and the bottom is sandy. South polar skua (*Stercorarius maccormicki*) have been observed in the area. Despite the lake’s proximity to the sea, no saltwater inflow is expected due to its elevated position. The surrounding area is devoid of vegetation.
64	Ornithologists Creek (Ornithologists)	27.12.2021	62°09’58” S 58°28’14” W	First described by Jurasz et al. [11] as the site of *B. gaini*, this is a small but deep pond located on glacial moraines. It likely connects to a temporary stream during snowmelt or heavy rains. The water is clear, and the rocky bottom is covered with microbial mats. Brown skua (*Stercorarius antarcticus*) nest nearby, and there are numerous lichens, mosses, and *Colobanthus quitensis* in the area.
66	Rakusa Point (Rakusa Point)	29.12.2021	62°10’01” S 58°27’37” W	A small, ephemeral puddle that dries out completely in summer. The bottom is sandy, with accumulations of bird feathers, driftwood deposited by tides or wind, and marine shrivelled macroalgae. The site is close to a gentoo penguin (*Pygoscelis papua*) colony, and penguins have been observed wallowing in the puddle. The copepod *Boeckella poppei* is abundant at this site. Sparse moss patches are present. Water column turbidity and depth vary with weather conditions.
72	Sphinx Glacier (Sphinx)	4.01.2022	62°11’38” S 58°26’20” W	A pond located near the shoreline of Admiralty Bay. The water is turbid, and the bottom is a mix of mud and stones. The surrounding area is covered by *Deschampsia antarctica* and mosses. Adélie penguins (*Pygoscelis adeliae*) have been observed entering the pond.
78	Turret Point (Turret)	5.01.2022	62°04’52” S 57°56’29” W	A small pond located on a hill. The water is clear, and the bottom is muddy, covered with mats of cyanobacteria and filamentous algae. Brown skua (*Stercorarius antarcticus*) nest nearby, and mosses and lichens are present in the area.

Basic physicochemical water parameters, including temperature, electrical conductivity, and pH, were measured using a Pro1030 multiparameter probe (YSI Inc. / Xylem Inc.) three times at each locality (Fig. 1C). Specimens of *B. gaini* were collected using a hand net during the austral summer season of 2021/2022 from eight sampling sites (Table 1). Preservation took place immediately after collection in the field. No live holding or transport prior to fixation was applied. Specimens collected from a single site were placed in labelled 50 mL Falcon tubes containing either prepared ethanol or formalin solution, depending on the intended downstream analysis. Five individuals from each site (a total of 40 specimens) were preserved in 96% ethanol and stored at − 20 °C for subsequent molecular analyses. Additional material fixed in formalin was used for microscopic examination of gut contents. Under laboratory conditions, preserved specimens were transferred to freshly prepared, labelled 2 mL tubes, with one specimen per tube. Due to technical limitations during field sampling, some water parameters were not recorded at the Hennequin and Rakusa sites.

Additionally, whenever possible, aerial photogrammetric surveys of the study area were conducted using an unmanned aerial vehicle (Inspire 2, DJI™) equipped with a digital camera (Fig. 1D). Notes were recorded for each site, including information on water turbidity (visual assessment: transparent or turbid), the presence of a bottom organic matter layer (present or absent), the presence of penguins, elephant seals, or skuas in the vicinity, and evidence of water flow (e.g. a stream flowing through the pond; constant flow, temporary flow, or static conditions).

### Microscopy-Based Analysis

Formalin-fixed specimens were used for analysis, including 10 individuals per site from Ecology, Hennequin, Ornithologists, Rakusa, and Sphinx, and five individuals per site from Viéville, Turret, and Llano. Specimens were rinsed in demineralized water, and their digestive tracts were dissected under a Delta Optical SZ-630T stereomicroscope using fine forceps. Microscopy-based analyses were performed exclusively on gut content samples free of gut tract material. Gut contents were transferred using either forceps or a pipette into microcentrifuge tubes containing 100 µL of prepared dispersant [25]. The contents of each tube were mixed using a pipette tip and then placed onto microscope slides. Prepared slides were examined under a Genetic Pro Trino (Delta Optical) compound microscope equipped with a Raspberry Pi HQ IMX477R 12.3 MPx camera.

The presence of various organisms was recorded for each sample, and photographic documentation of observed objects was obtained. Each digestive tract was examined individually, and microscopic data were recorded as presence–absence of dietary components per specimen; reported frequencies therefore represent the proportion of examined individuals per site in which a given component was observed. Organisms were identified using illustrations and taxonomic keys available in the literature [26–28], with particular attention given to taxa observed and reported by Paggi [5] and Janiec [29].

### Molecular Analyses

Ethanol-preserved specimens were rinsed in sterile TE buffer. For the purposes of downstream analyses, two intestinal fractions were distinguished: (i) gut contents, defined as luminal material representing ingested food and transient microorganisms, and (ii) gut tract, defined as the dissected intestinal tissue including the epithelium and associated peritrophic matrix after removal of luminal contents. Therefore, the analysed material comprised the midgut region together with its contents and the associated peritrophic matrix; foregut and hindgut structures were not analysed separately). Digestive tracts with their contents were dissected and rinsed again in TE buffer to separate gut contents from the gut tract itself using the stereomicroscope mentioned above. Each gut content was carefully examined for the presence of the peritrophic matrix. When present (in < 10% of specimens), the matrix was separated from the bulk gut contents. This separation was performed to allow comparative assessment of environmentally acquired, food-associated microbial communities (gut contents) versus those more closely associated with the lining of the gut tract. DNA extraction from the Hennequin site yielded insufficient material of adequate quality for downstream metabarcoding analyses. As a result, Hennequin samples were excluded from molecular analyses.

Cleaned gut tracts were placed into sterile microcentrifuge tubes, and samples from 5 individuals from each site were pooled to form one composite sample per location, ensuring sufficient material for subsequent procedures. A similar procedure was followed for the gut content suspensions obtained during the rinsing step; these were also pooled by site and processed as separate samples. Pooling was necessary due to the low biomass obtained from individual specimens. Consequently, each site is represented by a single composite sample, and within-site inter-individual biological variability could not be assessed. Gut content and gut tract samples originated from the same pooled individuals and therefore do not represent independent biological replicates. These compartments were treated as paired microhabitats within each site rather than as independent samples.

Genomic DNA was extracted from the samples, including both gut content and gut tract, using an enzymatic and detergent-based lysis protocol. Samples were centrifuged for 3 min at 10,000 rpm and resuspended in 100 µL of lysis buffer containing 50 mM Tris-HCl (pH 8.0) and 10 mM EDTA. To facilitate the breakdown of microbial cell walls, particularly those of Gram-positive bacteria, 10 µL of lysozyme (20 mg mL⁻¹; A&A Biotechnology) and 5 µL of mutanolysin (10,000 U mL⁻¹; A&A Biotechnology) were added to each sample. The mixture was incubated at 50 °C for 20 min to allow enzymatic digestion. Subsequently, 6 µL of 10% SDS and 5 µL of Proteinase K (20 mg mL⁻¹; A&A Biotechnology) were added to lyse residual cellular structures and digest proteins, followed by incubation at 50 °C for an additional 20 min. Following lysis and digestion, DNA was purified using a column-based DNA isolation kit (Clean-up Concentrator, A&A Biotechnology). The resulting DNA solution underwent quality control using a spectrophotometer (NanoPhotometer^®^ NP80, Implen GmbH).

The isolated DNA served as a template for PCR amplification using Illumina-compatible primers. The bacterial 16 S rRNA gene was amplified from both gut content and gut tract DNA using primers targeting the V3–V4 hypervariable regions (16S_V3-F: 5′-CCTACGGGNGGCWGCAG-3′; 16S_V4-R: 5′-GACTACHVGGGTATCTAATCC-3′), corresponding to positions 341–357 F and 785–805R, respectively, based on the *Escherichia coli* 16 S rRNA gene reference sequence [30]. Eukaryotic diversity was assessed exclusively in gut content samples by amplifying the V4 region of the 18 S rRNA gene using primers 18S_V4-F (5′-CCAGCAGCCGCGGTAATTCC-3′) and 18S_V4-R (5′-ACTTTCGTTCTTGATTAA-3′) (Choi and Park, 2020). Primers were synthesized with Illumina overhang adapter sequences to enable dual-indexed sequencing.

PCR amplification was performed using KAPA HiFi HotStart ReadyMix (Roche) in a total reaction volume of 25 µL containing 12.5 µL of ReadyMix, 0.2 µM of each primer, 10–50 ng of template DNA, and nuclease-free water. Thermal cycling conditions were as follows: initial denaturation at 95 °C for 3 min; 30 cycles of denaturation at 95 °C for 30 s, annealing at 58 °C for 30 s, and extension at 72 °C for 30 s; followed by a final extension at 72 °C for 5 min. Amplicon libraries were purified using AMPure XP beads (Beckman Coulter) and quantified with a Qubit fluorometer (Thermo Fisher Scientific). Dual indices and sequencing adapters were added in a second PCR using the Nextera XT Index Kit (Illumina), followed by additional purification. Libraries were normalized and pooled equimolarly based on qPCR quantification using the NEB Library Quant Kit (New England Biolabs). The pooled libraries were sequenced on an Illumina MiSeq platform (Illumina, San Diego, USA) at the DNA Sequencing and Synthesis Facility, Institute of Biochemistry and Biophysics, Polish Academy of Sciences. Sequencing was performed in paired-end mode (2 × 300 bp) using a MiSeq v3 (600-cycle) chemistry kit, enabling full overlap of the 16 S V3–V4 and 18 S V4 amplicons.

Extraction blanks (negative controls containing no added biological material) were included in each batch of nucleic acid extractions and processed alongside samples using identical reagents and protocols.

### Data Analysis

Illumina sequence reads were initially filtered using *fastp* (https://github.com/OpenGene/fastp) [31], quality-checked using the FastQC v.0.12.1 toolkit [32], and further processed using the QIIME2 pipeline (qiime2-amplicon-2024.10) [33]. Paired-end reads were merged and denoised using DADA2 implemented within QIIME2, and amplicon sequence variants (ASVs) were inferred using default parameters. Chimeric sequences were identified and removed during bioinformatic processing, and all downstream analyses were performed on non-chimeric ASVs. Contaminants were identified using the R package *decontam* with a combined prevalence- and frequency-based approach, incorporating both negative controls and DNA concentration measurements.

Sequences were taxonomically classified using a Bayesian naïve classifier pre-trained on the SILVA version 138.2 database optimized for the classification of Bacteria and Archaea [34], and the SILVA Eukaryotic 18 S database version 132. For the bacterial (16 S rRNA gene) dataset, non-target ASVs, including those classified as mitochondria, chloroplasts, Eukaryota, or unassigned at the domain level, were removed using the qiime taxa filter-table command. For the eukaryotic (18 S rRNA gene) dataset, sequences identified as Anostraca (host-derived) were removed using the same approach.

Downstream analyses were performed in the R v.4.3.3 environment. ASV count data were rarefied to an even sequencing depth using the rrarefy function in the *vegan* package. Rarefied ASV tables were used for alpha- and beta-diversity analyses. Relative abundances were calculated by normalizing ASV tables to a total sum of 1 using the decostand function (method = “normalize”) and were used for visualization purposes only. Rarefaction curves were generated from the ASV table using observed richness as a function of sequencing depth to evaluate sampling completeness across samples. Rarefied datasets were employed only for comparative analyses of relative community structure and diversity; no conclusions regarding absolute microbial abundances were drawn from these data. Rarefaction was applied to standardize sequencing depth across samples and enable direct comparison of diversity metrics. Although rarefaction has been criticized for discarding data and reducing statistical power, it remains widely used for ensuring comparability in datasets with uneven sampling depth. Given the limited number of composite samples and the focus on broad, qualitative patterns rather than precise estimation of absolute diversity, rarefaction was considered an appropriate and conservative approach in this study. Alternative normalization methods (e.g., variance-stabilizing transformations) may provide complementary insights and should be explored in future work.

Alpha diversity of bacterial communities was assessed using the Shannon diversity index, inverse Simpson index, and Faith’s phylogenetic diversity. Shannon and inverse Simpson indices were calculated using functions implemented in the *vegan* and *phyloseq* packages. Faith’s phylogenetic diversity was calculated using the pd function from the *picante* package based on a phylogenetic tree constructed from aligned 16 S rRNA gene sequences. Given the compositional nature of amplicon data and the use of pooled samples, we focused on diversity metrics (e.g., Shannon, inverse Simpson, Faith’s PD) that integrate both richness and evenness and are less sensitive to undersampling of rare taxa.

Beta diversity was assessed using Bray–Curtis dissimilarities calculated from rarefied ASV abundance tables with the vegdist function in the *vegan* package. Hierarchical clustering (UPGMA) was performed using the hclust function in R with the average linkage method based on Bray–Curtis dissimilarity matrices. Principal coordinates analysis (PCoA) was performed using the cmdscale function and visualized using *ggplot2*. Statistical differences in community composition were tested using permutational multivariate analysis of variance (PERMANOVA) implemented with the adonis2 function in the *vegan* package (9,999 permutations). Homogeneity of multivariate dispersions was assessed using PERMDISP via the betadisper function. The correspondence between bacterial (16 S rRNA) and eukaryotic (18 S rRNA) community structures was evaluated using Procrustes analysis, and the correlation between distance matrices was assessed with a Mantel test.

Differences in Bray–Curtis dissimilarities calculated within ponds and between ponds were tested using a Wilcoxon rank-sum test implemented with the wilcox.test function in R. The proportion of shared ASVs was calculated as the number of ASVs detected in gut tract samples that were also present in gut content samples, divided by the total number of gut tract–associated ASVs, and calculated separately for each pond; a global pooled proportion was additionally calculated across all samples. Relative abundances of shared ASVs were calculated from normalized ASV tables and summarized using boxplots.

Shared gut tract–associated ASVs among sampling sites were identified based on binary presence–absence matrices derived from ASV tables and visualized using UpSet plots.

To assess the influence of environmental conditions on gut tract–associated bacterial community composition, Bray–Curtis dissimilarities were calculated from rarefied gut tract ASV tables and visualized using PCoA. Physicochemical variables measured at the sampling sites (pH, electrical conductivity, and temperature) were analysed separately. Mean values of physicochemical parameters were used for subsequent statistical analyses. For visualization purposes, samples were grouped according to whether the measured value of a given physicochemical parameter was below or above the mean across sampling sites. Environmental variables were categorized into values above and below the mean to facilitate visualization of patterns in ordination analyses. This approach was used as a descriptive simplification of continuous gradients and should not be interpreted as reflecting discrete environmental categories. Differences in community composition between groups were tested using PERMANOVA, and homogeneity of multivariate dispersions was assessed using PERMDISP. Grouping thresholds were defined using mean values of environmental variables, as this approach provided more stable group structure and avoided heterogeneity of dispersion among groups, which is critical for PERMANOVA interpretation.

Generalized additive models (GAMs) were used to evaluate potentially non-linear relationships between water physicochemical variables and bacterial alpha diversity. The Shannon diversity index was used as the response variable, while pH, conductivity, and temperature were included as explanatory variables in separate models. GAMs were fitted using the *mgcv* package in R. Model significance was evaluated using approximate F-tests, and model performance was assessed using adjusted R² values.

## Results

### Study Sites and Environmental Heterogeneity

The studied ponds spanned a wide range of spatial and physicochemical conditions across the coastal zone of King George Island (Fig. 1A, B; Supplementary Table S1). Pond surface area varied by nearly two orders of magnitude, ranging from approximately 12.5 to 47 419.6 m² (mean 7 617.4 m²).

Water physicochemical parameters differed markedly among sites (Fig. 1C). Water pH ranged from slightly acidic to alkaline (6.4–8.8, mean 7.8), with the lowest values recorded at the Viéville proglacial pond and the highest at Llano. Water temperature averaged 5.8 °C, with the warmest conditions observed at Ornithologists (8.2 °C) and the coldest at the Viéville site (3.4 °C). Electrical conductivity exhibited substantial variability among ponds, spanning from 149 µS cm⁻¹ at Viéville to 776 µS cm⁻¹ at Ecology.

The ponds were distributed along pronounced altitudinal and coastal gradients (Fig. 1B; Supplementary Table S1). Ornithologists and Turret were situated at higher elevations (50 and 45 m a.s.l., respectively), whereas Viéville and Rakusa occurred at 10 m a.s.l., and Llano, Ecology, and Sphinx were located at 5 m a.s.l. Viéville was positioned directly at the glacier terminus, while the remaining ponds were located farther from glaciers, at an average distance of 631 ± 126 m. In contrast, most sites were situated close to the coastline (< 45 m), with the exception of Ornithologists and Turret, which were located substantially farther inland (360 and 238 m from the coast, respectively).

Measurements for selected physicochemical parameters could not be obtained at some sites (Rakusa and Hennequin) due to technical or logistical constraints.

### Gut content Composition Assessed By Microscopy and Metabarcoding

**Fig. 2:**
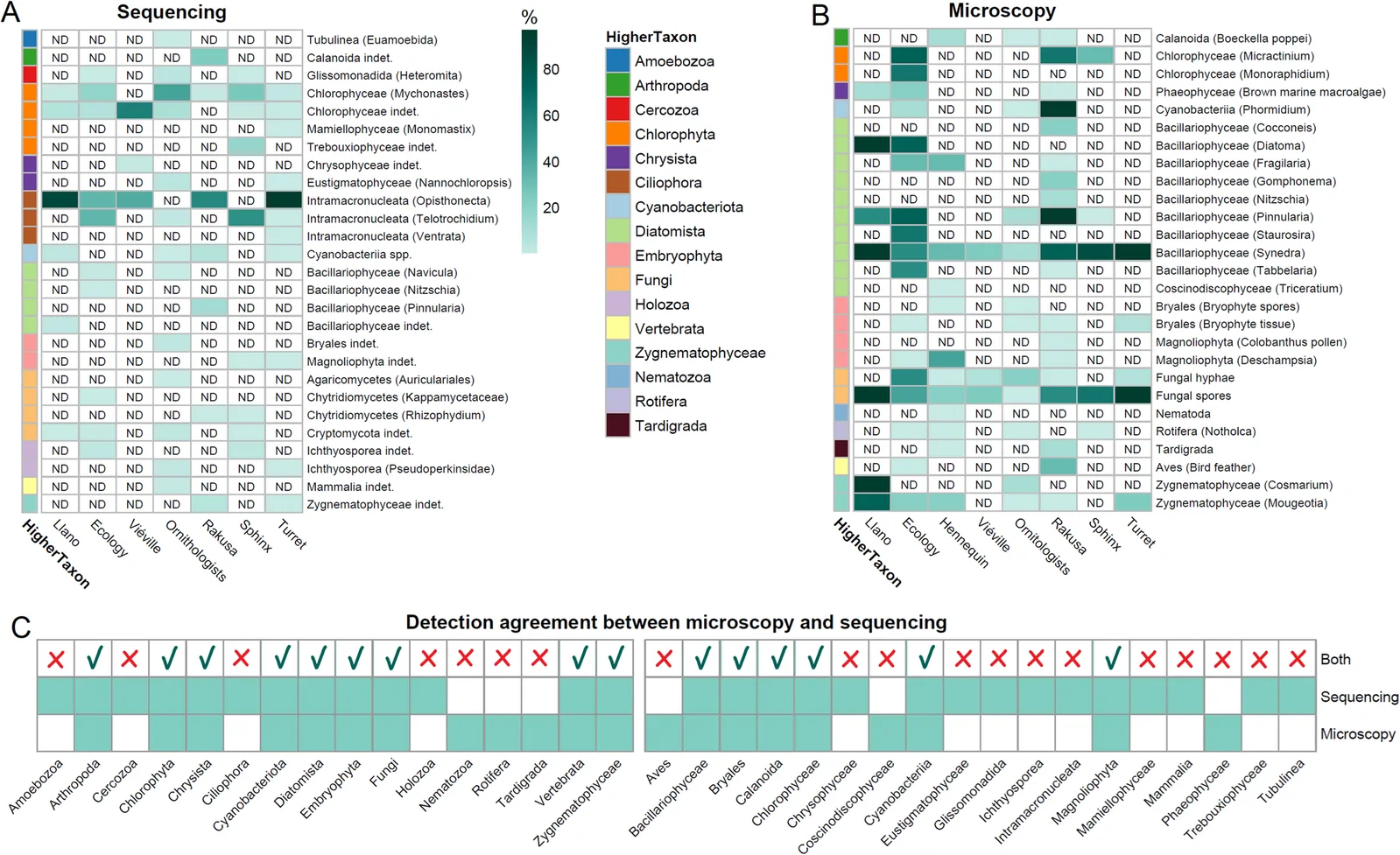
Composition and diversity of gut contents in Antarctic *Branchinecta gaini*. (**A**) Heatmap showing the relative composition of gut contents based on high-throughput sequencing, including primarily eukaryotic taxa identified using the 18 S rRNA gene (V4 region), with cyanobacterial sequences (16 S rRNA gene) additionally included to facilitate comparison with microscopy-based observations. Values represent relative sequence abundance (%). (**B**) Heatmap showing the composition of gut contents assessed by light microscopy, expressed as the percentage frequency of occurrence of organismal groups across sampling sites. Values represent frequency of occurrence (% of individuals per site in which a given component was detected). Frequency of occurrence was calculated as the proportion of analysed individuals per site in which a given component was detected (**C**) Presence–absence matrix comparing gut content components detected by sequencing and light microscopy. The classification scheme shown in the legend refers to higher-level taxonomic groups and applies to both heatmaps

Sequencing-based analysis of gut contents revealed pronounced differences in taxonomic composition among ponds, with clear site-specific patterns observed in pooled samples (Fig. 2A). Although all ponds contained a mixture of photosynthetic, heterotrophic, and metazoan-associated taxa, their relative contributions varied markedly. Rarefaction curves indicated sufficient sequencing depth for both 16 S and 18 S datasets (Supplementary Figs. S1 and S2).

A substantial proportion of recovered 18 S rRNA gene amplicons across all samples was assigned to Anostraca. These sequences were not included in Fig. 2A, as it could not be determined whether they originated from host DNA or from ingested conspecific material. Gut contents from Llano, Rakusa, and Turret were dominated by ciliates of the class *Intramacronucleata (Opisthonecta)*, which accounted for 89.5%, 55.2%, and 96.6% of recovered sequences, respectively. In these ponds, other taxonomic groups contributed only marginally to the overall composition. In contrast, Ecology and Sphinx exhibited more heterogeneous profiles, characterized by a substantial contribution of both ciliates and chlorophytes. In Ecology, *Opisthonecta* (34.0%) co-occurred with *Telotrochidium* (34.1%) and chlorophytes, particularly *Chlorophyceae* (ca. 24% combined). Similarly, Sphinx showed a mixed assemblage dominated by *Telotrochidium* (51.8%) alongside chlorophytes, including *Mychonastes* (26.6%) and *Trebouxiophyceae* (16.5%). Ornithologists displayed a distinct gut content composition, with chlorophytes representing the dominant component, primarily *Mychonastes* (43.2%) and *Chlorophyceae indet.* (9.3%). This site also showed a relatively high contribution of heterotrophic protists, including *Glissomonadida* (4.5%), as well as minor but diverse contributions from eustigmatophytes (*Nannochloropsis*), fungi, ichthyosporeans, and metazoan-associated sequences. Viéville was characterized by a unique profile dominated by *Chlorophyceae indet.* (59.6%) and ciliates (*Opisthonecta*, 38.8%), with only minor contributions from other groups. Notably, diatom-associated sequences were absent from Viéville gut contents, distinguishing this site from several other ponds. Diatoms (*Bacillariophyceae*) were detected only at selected sites and always at low relative abundances, with *Navicula* and *Nitzschia* detected in Ecology and Ornithologists, *Pinnularia* in Rakusa, and unidentified diatoms in Llano.

Cyanobacterial sequences were present at low to moderate levels in Llano (3.1%), Rakusa (6.0%), and Ornithologists (0.3%), but were absent from several other sites. Metazoan-associated sequences were rare and highly site-specific, including calanoid copepods detected exclusively in Rakusa and vertebrate-associated sequences detected only in Ornithologists. Overall, no single taxonomic group dominated gut contents across all ponds, and each site exhibited a distinct sequencing-based gut content profile at the composite level.

Light microscopy revealed a taxonomically diverse gut content composition with pronounced differences in the frequency of occurrence of individual components among ponds (Fig. 2B). In contrast to sequencing-based profiles, microscopy emphasized the widespread occurrence of benthic and periphytic taxa, as well as particulate and metazoan material, across individual hosts. Diatoms (*Bacillariophyceae*) were among the most frequently observed components of gut contents and occurred across all ponds, although their frequency varied among sites. *Synedra* was the most consistently detected diatom genus, occurring in a high proportion of individuals at most sites, including Llano, Rakusa, Sphinx, and Turret. Other diatom taxa, such as *Pinnularia*, *Diatoma*, *Fragilaria*, and *Staurosira*, exhibited marked site-specific patterns, with high frequencies in Ecology and Rakusa but limited or no occurrence in Viéville. Green algae (*Chlorophyta*) were also commonly observed but showed spatial heterogeneity. *Micractinium* and *Monoraphidium* occurred frequently in Ecology and Rakusa, while being absent or rare in several other ponds. Zygnematophytes (*Cosmarium* and *Mougeotia*) were predominantly detected in Llano and, to a lesser extent, Ecology and Turret, but were largely absent from Viéville and Sphinx.

Cyanobacteria (*Phormidium*) were detected at high frequency in Rakusa, where they occurred in all examined individuals, but were rare or absent at most other sites. Chrysophytes and phaeophyte material were observed sporadically and at low frequencies, restricted to a small subset of ponds. Metazoan components were detected infrequently but consistently across sites. Copepods (*Boeckella poppei*) occurred in a limited number of individuals at Hennequin, Ornithologists, and Rakusa. Rotifers (*Notholca*), nematodes, and tardigrades were observed only sporadically, while vertebrate-derived material (bird feathers) was detected exclusively at Ecology and Rakusa. Non-algal eukaryotic material, including fungal hyphae and spores, was commonly observed across ponds, with fungal spores occurring at high frequencies in Llano and Turret and moderate frequencies at most other sites. Bryophyte tissue and spores, as well as pollen and fragments of vascular plants, were detected sporadically, primarily in Ecology, Hennequin, and Rakusa. Overall, microscopy-based observations revealed high within-site variability in gut content composition and highlighted spatial heterogeneity among ponds, with no single taxonomic component occurring at uniformly high frequencies across all sites. In addition, fragments of *Branchinecta gaini* were observed in gut contents of individuals from all sampled ponds, indicating the ubiquitous presence of conspecific material across sites.

A presence–absence comparison of gut content components detected by sequencing and light microscopy at two taxonomic resolutions was applied: higher-level taxa and selected lower-level taxonomic groups (Fig. 2C). At the level of higher taxonomic groups, several components were detected by both methods at multiple ponds, including *Chlorophyta*, *Diatomista*, Fungi, and *Zygnematophyceae*, although not uniformly across all sites. In contrast, pronounced method-specific detection patterns were observed for other groups. *Ciliophora* (*Intramacronucleata*), *Cercozoa*,* Amoebozoa*, and *Holozoa* were detected exclusively by sequencing, whereas several metazoan groups, including *Rotifera*, *Tardigrada*, and *Nematozoa*, were detected only by microscopy. Comparison at a finer taxonomic resolution further emphasized the partial overlap between methods. Sequencing detected a broader range of protistan and algal lineages, including *Glissomonadida*, *Ichthyosporea*, *Eustigmatophyceae*, *Mamiellophyceae*, *Trebouxiophyceae*, and *Tubulinea*, which were not observed microscopically. Conversely, microscopy uniquely detected several morphologically distinctive components, such as *Coscinodiscophyceae*, *Phaeophyceae*, and avian-derived material (Aves), which were absent from sequencing-based records. Despite these discrepancies, some lower-level taxa, including *Bacillariophyceae*, *Chlorophyceae*, *Cyanobacteriia*, and *Bryales*, were detected by both methods at multiple sites, although rarely in the same combination across all ponds. No pond showed complete concordance between sequencing and microscopy across either taxonomic resolution.

**Fig. 3:**
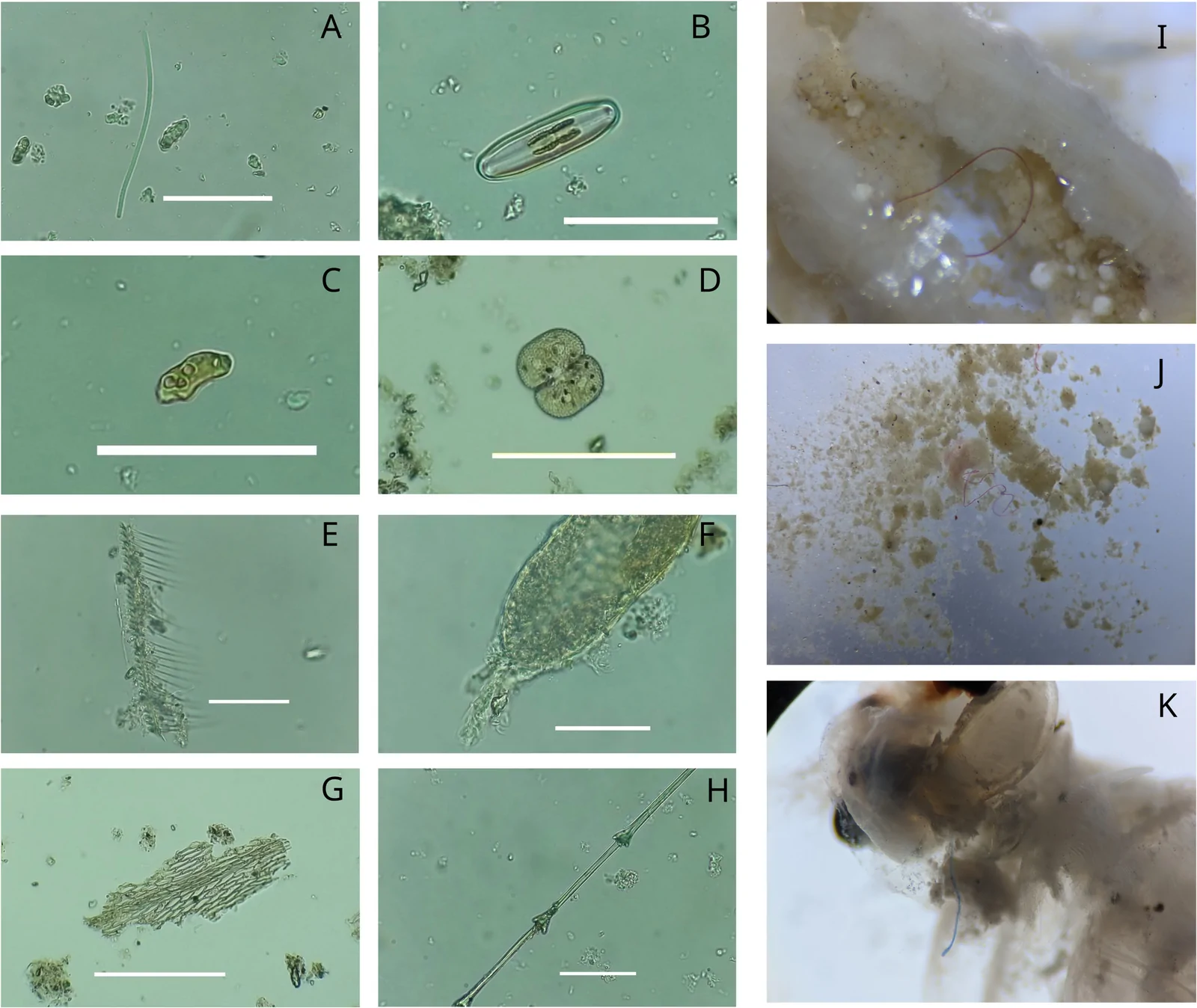
Representative food items, ingested debris, and microplastic particles observed in *Branchinecta gaini*. (**A**–**H**) Light microscopy images of gut contents showing: (**A**) cyanobacteria, (**B**) a diatom, (**C**) fungal spores, (**D**) green algae, (**E**) an exopodite fragment of *B*. *gaini*, (**F**) a tardigrade, (**G**) moss tissue, and (**H**) bird feather/down. White scale bars in panels A–H represent 50 µm. (**I–K**) Stereomicroscopy images of dissected *B*. *gaini* individuals showing ingested microplastic fibres: (**I**) a red fibre, (**J**) a red fibre accompanied by additional small particles, and (**K**) a blue fibre protruding from the oral region

Microscopic examination of gut contents revealed a wide range of ingested biological material and particulate debris, consistent with the taxonomic patterns summarized in Fig. 2. Representative examples of food items, detrital material, and microplastic fibers observed during light and stereomicroscopy are shown in Fig. 3.

### Bacterial Community Diversity and Composition in Gut Microcompartments 

**Fig. 4:**
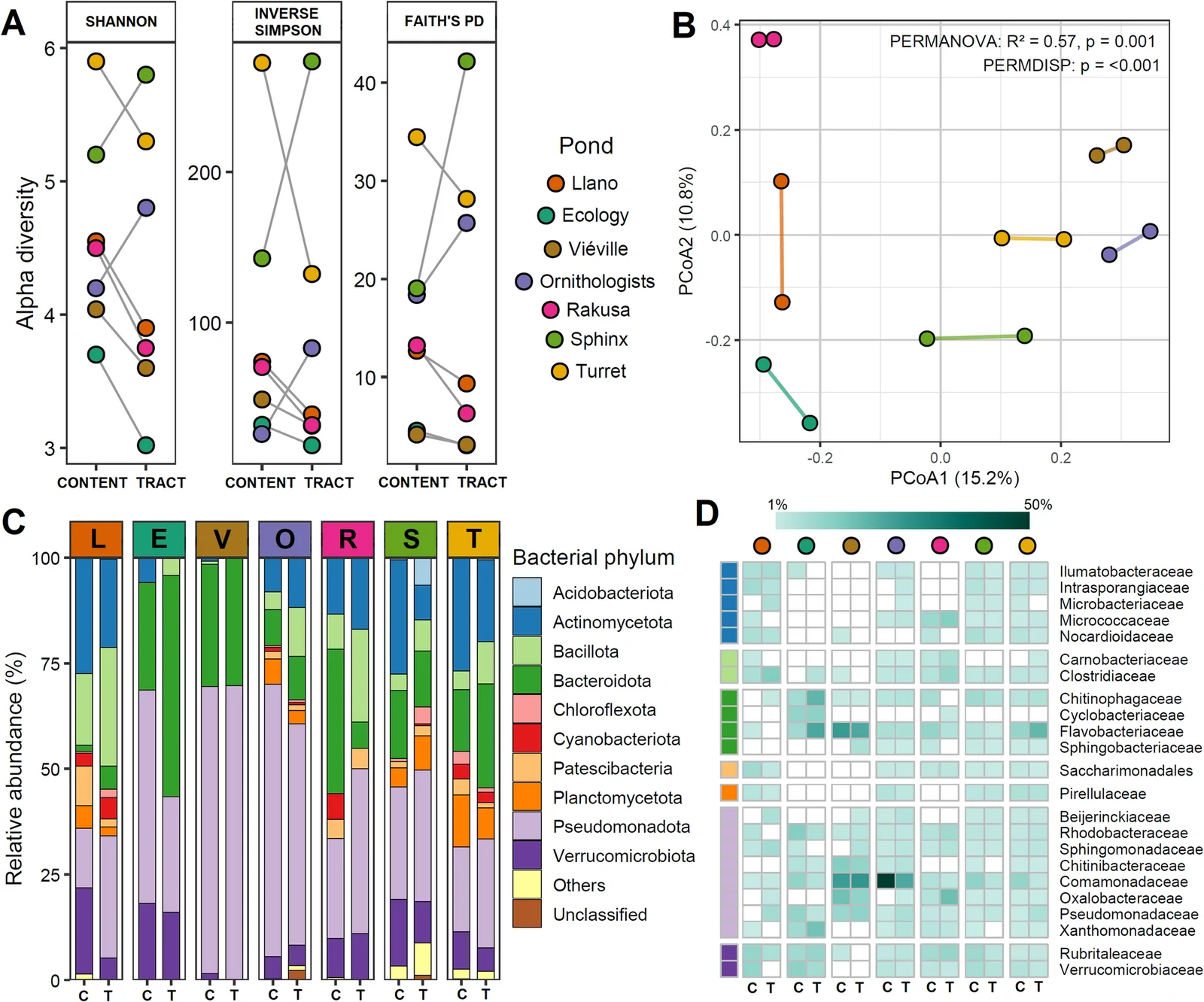
Bacterial community diversity and composition in two gut microcompartments of *Branchinecta gaini* based on 16 S rRNA gene sequencing. (**A**) Alpha diversity metrics (Shannon index, inverse Simpson index, and Faith’s phylogenetic diversity) comparing gut contents and gut tract across seven ponds. Points represent individual samples, colours indicate sampling sites, and grey lines connect paired samples from the same individual. (**B**) Principal coordinates analysis (PCoA) of Bray–Curtis dissimilarities showing differences in bacterial community composition between gut contents and gut tract. Results of PERMANOVA (R² and *p-value*) and PERMDISP are shown. (**C**) Relative abundance of bacterial phyla in gut contents (C) and gut tract (T) across sampling sites. Letters above the bars represent initials of pond names (L – Llano, E – Ecology, V – Viéville, O – Ornithologists, R – Rakusa, S – Sphinx, T – Turret). (**D**) Heatmap showing the relative abundance of dominant bacterial families (1–50%) in gut contents (C) and gut tract (T) across sampling sites. Sampling sites are indicated by coloured circles above the heatmap

Alpha diversity of bacterial communities varied substantially in pooled samples both between gut microcompartments and among ponds (Fig. 4A). Across all samples, Shannon diversity ranged from 3.0 to 5.9, inverse Simpson diversity from approximately 18.6 to 273.3, and Faith’s phylogenetic diversity from 3.0 to 42.2, indicating pronounced heterogeneity in bacterial diversity across sites. The correspondence between bacterial (16 S rRNA) and eukaryotic (18 S rRNA) community structures was weak, as indicated by Procrustes analysis and a non-significant Mantel test (Supplementary Fig. S3). Differences between gut contents and gut tract were pond-specific. In Llano, Ecology, Viéville, and Ornithologists, bacterial diversity tended to be higher in gut contents than in the gut tract, with Shannon indices decreasing from 4.0 to 4.6 in gut contents to 3.0–3.9 in the gut tract, and inverse Simpson diversity declining from 32 to 74 to 19–40. Similarly, Faith’s phylogenetic diversity was reduced in the gut tract at these sites, for example from 12.7 to 9.3 in Llano and from 13.2 to 6.3 in Ornithologists. In contrast, Rakusa and Sphinx exhibited higher bacterial diversity in the gut tract compared to gut contents. At Rakusa, Shannon diversity increased from 4.2 in gut contents to 4.8 in the gut tract, accompanied by a threefold increase in inverse Simpson diversity (from 26.2 to 83.0) and higher phylogenetic diversity (18.4 to 25.7). The highest compartmental contrast was observed at Sphinx, where inverse Simpson diversity nearly doubled from 142.6 in gut contents to 273.3 in the gut tract, and Faith’s phylogenetic diversity increased from 19.0 to 42.2.

Marked differences in alpha diversity were also observed among ponds irrespective of gut compartment. Turret and Sphinx consistently harboured the most diverse bacterial communities, with high Shannon (≥ 5.2), inverse Simpson (> 130), and Faith’s PD (> 28) values in both gut contents and gut tract. In contrast, Ecology and Viéville showed comparatively low bacterial diversity across compartments, particularly in terms of phylogenetic diversity, which remained below 5 in gut contents and below 3.1 in the gut tract. Overall, alpha diversity patterns indicate that pond identity appears to influence bacterial diversity, with compartment-specific differences superimposed on pronounced between-pond variability.

Beta diversity analysis based on Bray–Curtis dissimilarities revealed differences in bacterial community composition between gut contents and gut tract, as well as spatial structuring among ponds as observed at the composite level (Fig. 4B). Principal coordinates analysis (PCoA) showed a clear separation of samples according to gut microcompartment, with paired samples from the same pond connected by lines. PERMANOVA indicated that gut microcompartment explained a substantial proportion of variation in bacterial community composition (R² = 0.57, *p* = 0.001) although this should be interpreted cautiously given pooling. However, this pattern was accompanied by significant heterogeneity of dispersion between groups, as indicated by PERMDISP (*p* < 0.001), suggesting that differences in within-group variability also contributed to the observed separation. In addition to compartmental effects, samples clustered by pond of origin, indicating site-specific signatures in bacterial community composition, suggesting site-associated patterns rather than strong inferred effects.

Ponds such as Sphinx and Turret formed relatively compact clusters, whereas other sites, including Rakusa and Ornithologists, showed greater dispersion in ordination space, reflecting higher variability among samples from these locations. Overall, beta diversity patterns demonstrate that bacterial community composition differed consistently between gut contents and gut tract, while also being shaped by pond-specific factors, with compartmental differences superimposed on pronounced spatial heterogeneity.

Bacterial community composition at the phylum level in pooled samples varied among ponds and between gut contents and gut tract (Fig. 4C). Across all sites, communities were dominated by *Pseudomonadota*, *Bacteroidota*, *Actinomycetota*, *Bacillota*, and *Verrucomicrobiota*, with substantial variation in their relative contributions.

Some ponds (e.g., Viéville and Ornithologists) were characterized by strong dominance of *Pseudomonadota*, whereas others (e.g., Llano, Rakusa, Sphinx, and Turret) exhibited more even and taxonomically heterogeneous profiles. Differences between gut contents and gut tract were observed across ponds but were not uniform. In general, *Bacillota* and *Pseudomonadota* tended to be relatively more abundant in the gut tract, while *Verrucomicrobiota* and *Patescibacteria* were more prominent in gut contents. Low-abundance phyla (e.g., *Cyanobacteriota*, *Chloroflexota*) showed site-specific variation.

Overall, phylum-level composition reflected pond-associated patterns, with compartmental differences superimposed but not consistent across all sites.

At the family level, bacterial community composition differed between gut contents and gut tract and varied among ponds (Fig. 4D). A limited number of dominant families accounted for most of the observed variation. It should be noted that all abundance values are relative and do not reflect absolute cell numbers. Consequently, differences in relative abundance do not necessarily correspond to differences in absolute abundance, and may also be influenced by variation in rRNA gene copy number.

Within *Pseudomonadota*, *Comamonadaceae* were consistently among the most abundant families across multiple sites, with notable contributions also from *Rhodobacteraceae*, *Sphingomonadaceae*, and *Pseudomonadaceae*. Within *Bacteroidota*, *Flavobacteriaceae* and *Chitinophagaceae* were prominent, often showing site-specific enrichment. *Actinomycetota* were represented by several moderately abundant families (e.g., *Ilumatobacteraceae*, *Intrasporangiaceae*, *Nocardioidaceae*), while *Bacillota*-associated families, particularly *Clostridiaceae*, tended to be relatively more abundant in the gut tract.

*Verrucomicrobiota*-associated families (e.g., *Rubritaleaceae*, *Verrucomicrobiaceae*) also contributed substantially but showed variable distribution across ponds and compartments.

Overall, family-level patterns indicate that differences between gut contents and gut tract were driven by shifts in a small number of dominant families and were not consistent across all sites.

### Dissimilarity and Taxonomic Overlap Between Gut Content– and Gut Tract–Associated Bacterial Communities

**Fig. 5:**
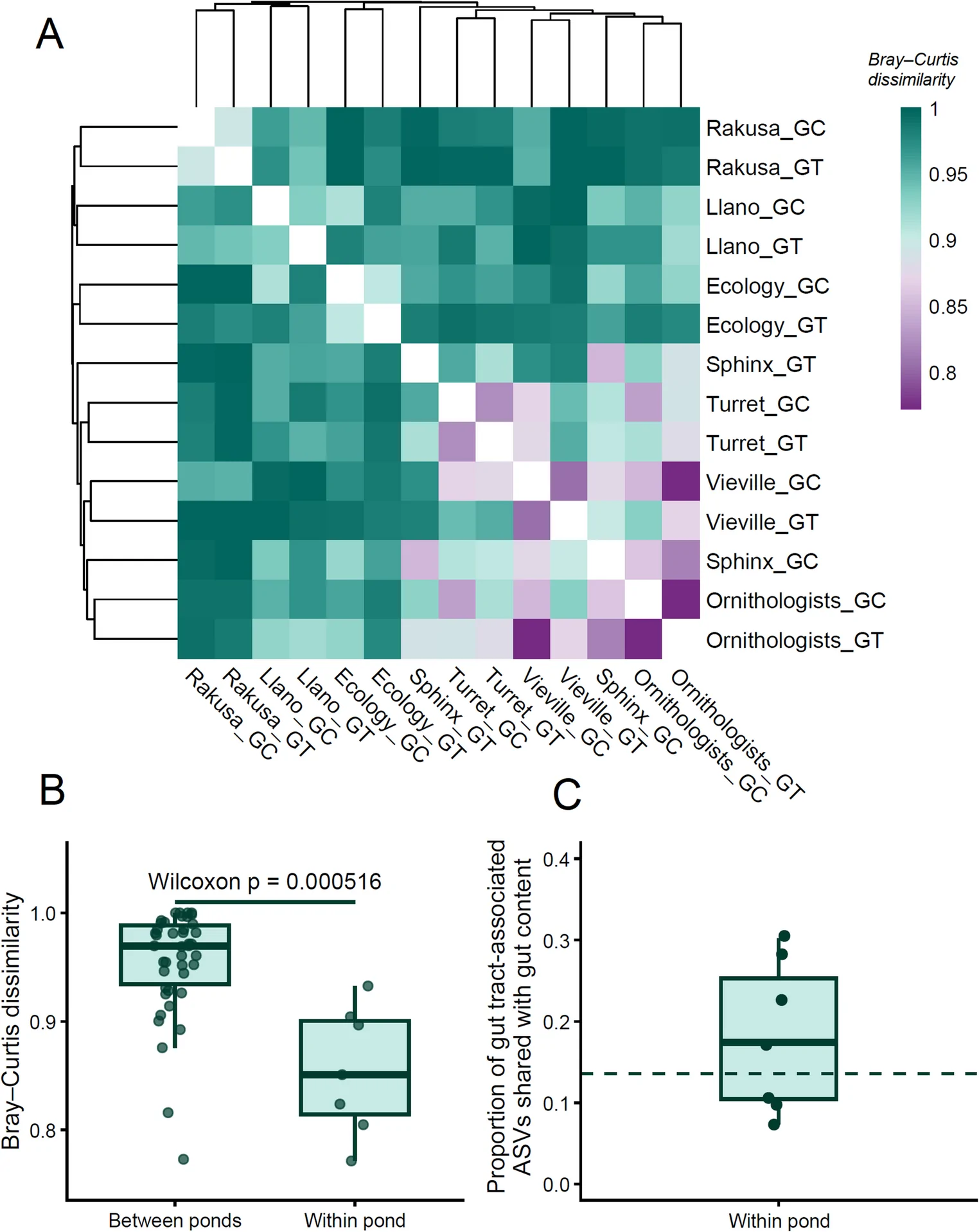
Dissimilarity and overlap between bacterial communities associated with gut contents and gut tract of *Branchinecta gaini*. (**A**) Hierarchically clustered heatmap of pairwise Bray–Curtis dissimilarities between bacterial communities from gut contents (GC) and gut tract (GT) across all sampling sites. Dendrograms indicate clustering of samples based on community composition. (**B**) Comparison of Bray–Curtis dissimilarities calculated between ponds and within ponds. Boxplots show median and interquartile range; points represent individual pairwise comparisons. Statistical significance was assessed using a Wilcoxon rank-sum test. (**C**) Proportion of gut tract–associated ASVs shared with gut content communities. Boxplot shows the distribution of the proportion of shared ASVs calculated separately for each pond (*n* = 7). Points represent individual ponds. The dashed horizontal line indicates the global pooled proportion of shared ASVs calculated across all samples (13.6%), shown for reference

Pairwise comparisons of bacterial community composition at the pooled level revealed dissimilarity between gut content– and gut tract–associated communities across ponds (Fig. 5). Hierarchically clustered Bray–Curtis dissimilarities showed that most samples clustered primarily by pond of origin rather than by gut microcompartment, while gut content and gut tract samples from the same pond still remained highly dissimilar (Fig. 5A). These patterns were not consistent across all ponds and in some cases appeared to be driven by a subset of sites.

Quantitative comparison of Bray–Curtis dissimilarities confirmed that bacterial communities differed more between ponds than within ponds (Fig. 5B). Median Bray–Curtis dissimilarity values were higher for between-pond comparisons (median approximately 0.96) than for within-pond comparisons between gut contents and gut tract (median approximately 0.85). This difference was statistically significant (Wilcoxon rank-sum test, *p* = 0.000516), indicating that pond identity explained a larger proportion of community dissimilarity than gut microcompartment alone. These patterns suggest spatial structuring but should not be interpreted at the individual level.

Despite this, overlap between gut content– and gut tract–associated communities within ponds was limited (Fig. 5C). The proportion of gut tract–associated ASVs shared with gut content communities varied among ponds but remained consistently low, ranging from approximately 7% to 30%, with a median of around 17%. The pooled proportion of shared ASVs across all samples was 13.6%, indicating that only a small fraction of gut tract–associated bacterial diversity was also detected in gut contents. Together, these results demonstrate that bacterial communities associated with the gut tract are largely distinct from those present in gut contents, while both compartments are structured by pond-specific factors.

**Fig. 6:**
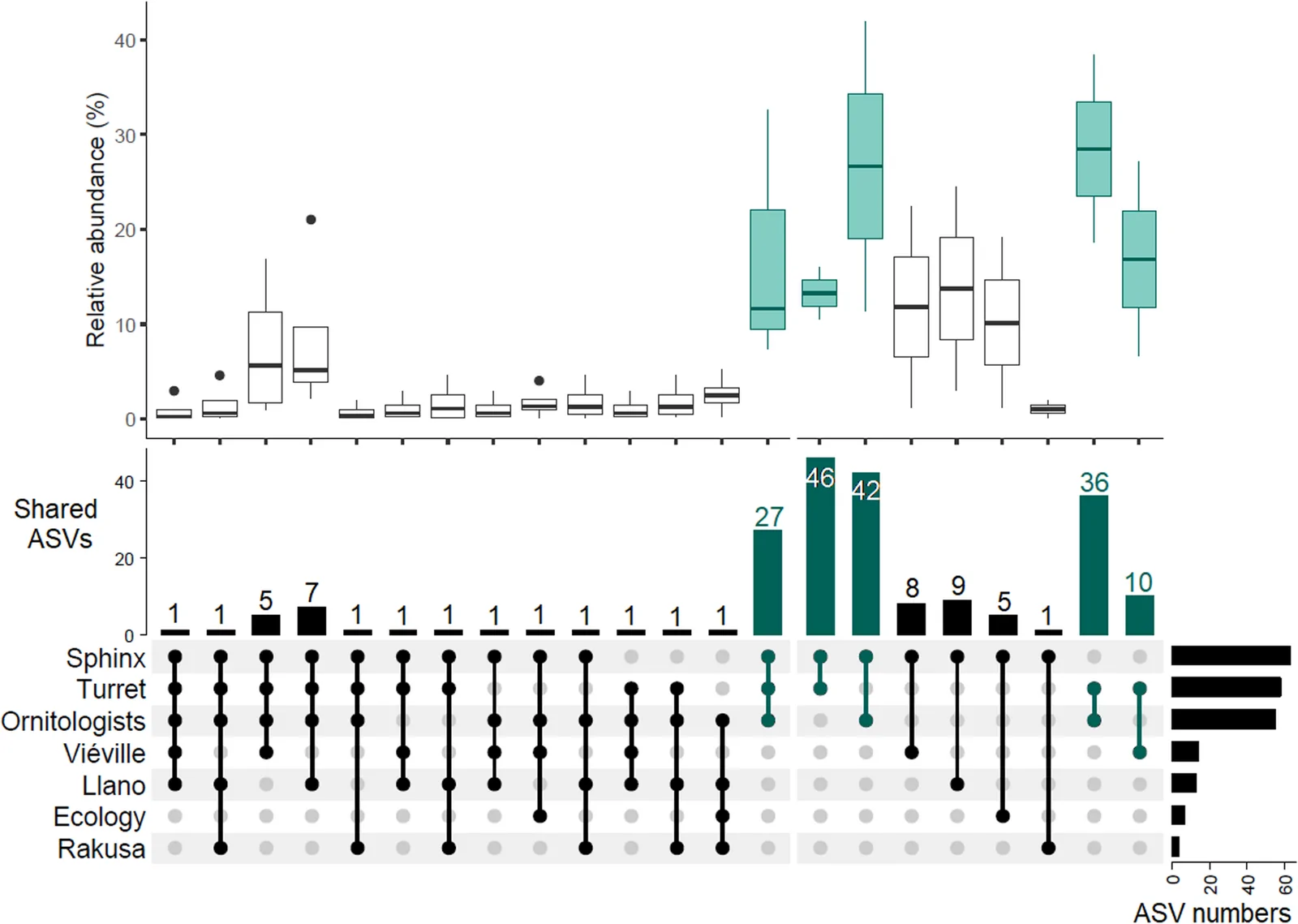
Shared gut tract–associated bacterial ASVs across sampling sites and their relative abundance. The lower panel shows an UpSet plot illustrating the number of shared gut tract–associated ASVs among sampling sites. Filled circles connected by vertical lines indicate specific site combinations, and bars above represent the number of ASVs shared among the indicated sites. Horizontal bars on the right show the total number of gut tract–associated ASVs detected at each site. The upper panel shows the relative abundance (%) of these shared ASVs within gut tract samples, summarized as boxplots across sites. Boxes represent the interquartile range with medians indicated, and whiskers denote the range excluding outliers. Boxplots highlighted in green indicate shared ASVs for which the median relative abundance across samples exceeded 10%

Patterns of ASV sharing were structured by pond identity (Fig. 6). The largest sets of shared gut tract–associated ASVs were observed among Sphinx, Turret, and Viéville, both in pairwise and multi-site combinations. In particular, ASVs shared between Sphinx and Turret, as well as those shared among Sphinx, Turret, and Viéville, represented the largest ASV intersections in the UpSet analysis. Additional high-sharing combinations involved Ornithologists together with Sphinx and Turret, indicating partial overlap among these sites. In contrast, ponds such as Rakusa and Ecology contributed relatively few shared ASVs and were dominated by site-specific ASVs, resulting in small or absent intersections with other ponds. Thus, ASV sharing was uneven across sites, with a subset of ponds accounting for the majority of multi-site shared ASVs. Notably, the ponds that shared the highest numbers of gut tract–associated ASVs corresponded to those that clustered more closely in Bray–Curtis–based analyses (Fig. 5A), indicating concordance between taxonomic overlap and overall community similarity.

### Environmental Drivers of Gut Bacterial Communities

**Fig. 7:**
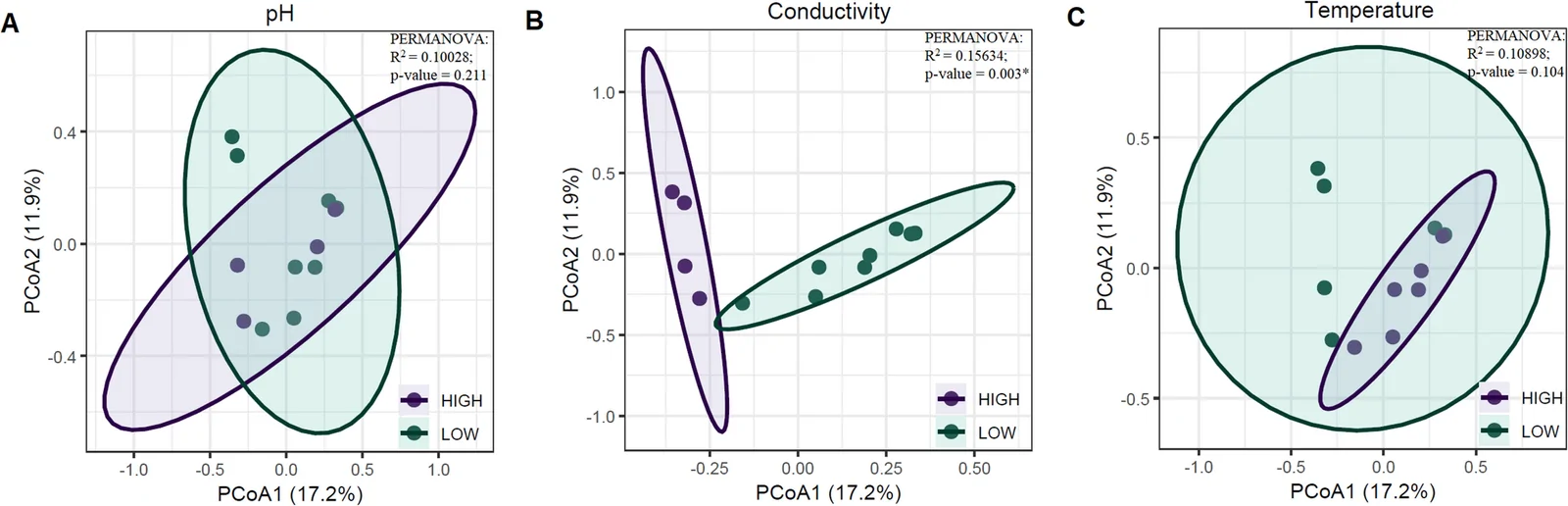
Associations between environmental conditions and gut bacterial community composition of *Branchinecta gaini*. (**A**–**C**) Principal coordinates analysis (PCoA) based on Bray–Curtis dissimilarities of 16 S rRNA gene ASV data from gut tract samples, shown separately for pH (A), conductivity (**B**), and temperature (**C**). Samples are grouped according to environmental conditions at the sampling sites, categorized as values below and above the mean for each physicochemical parameter for visualization purposes only. Differences in community composition between groups were evaluated using PERMANOVA; corresponding R² and p-values are shown on the plots. Homogeneity of multivariate dispersions was assessed using PERMDISP and was not significant in any comparison. Given the limited number of sites and pooled sampling design, these analyses should be interpreted as exploratory. Each point represents a composite sample, and gut microcompartments are not independent biological replicates. Axis scales differ between panels to optimize visualization of the ordination structure in each case. Mean pH = 7.8, mean conductivity = 370.4 µS cm⁻¹, mean temperature = 5.8 °C

Principal coordinates analyses based on Bray–Curtis dissimilarities revealed variable effects of environmental parameters on gut bacterial community composition (Fig. 7; Supplementary Tables S3 and S4), however, these analyses should be considered exploratory. Due to missing environmental data for some sites (e.g., Rakusa), the number of samples included in these analyses was reduced (*n* = 6–7 depending on the variable). When samples were grouped according to pH values below and above the site mean, no significant separation of bacterial communities was observed (PERMANOVA R² = 0.100, *p* = 0.211; Fig. 7A), indicating that pH explained only a small proportion of variation in gut-associated bacterial assemblages.

In contrast, electrical conductivity showed a significant association with bacterial community composition (Fig. 7B). Samples from sites with lower and higher conductivity values formed distinct clusters in ordination space, and PERMANOVA indicated that conductivity explained a moderate proportion of variation (R² = 0.156, *p* = 0.003). However, this pattern should be interpreted cautiously given the limited sample size and composite nature of the data. Differences in multivariate dispersion were not significant, suggesting that the observed separation likely reflected differences in community composition rather than unequal within-group variability. Temperature explained a smaller proportion of variation in community composition (PERMANOVA R² = 0.109, *p* = 0.104; Fig. 7C). Although some separation between low- and high-temperature groups was visible in ordination space, this pattern was weak and not supported by statistical significance.

Overall, these analyses indicate that conductivity was associated with variation in gut bacterial community composition at the population level, whereas pH and temperature explained relatively limited variation.

**Fig. 8:**
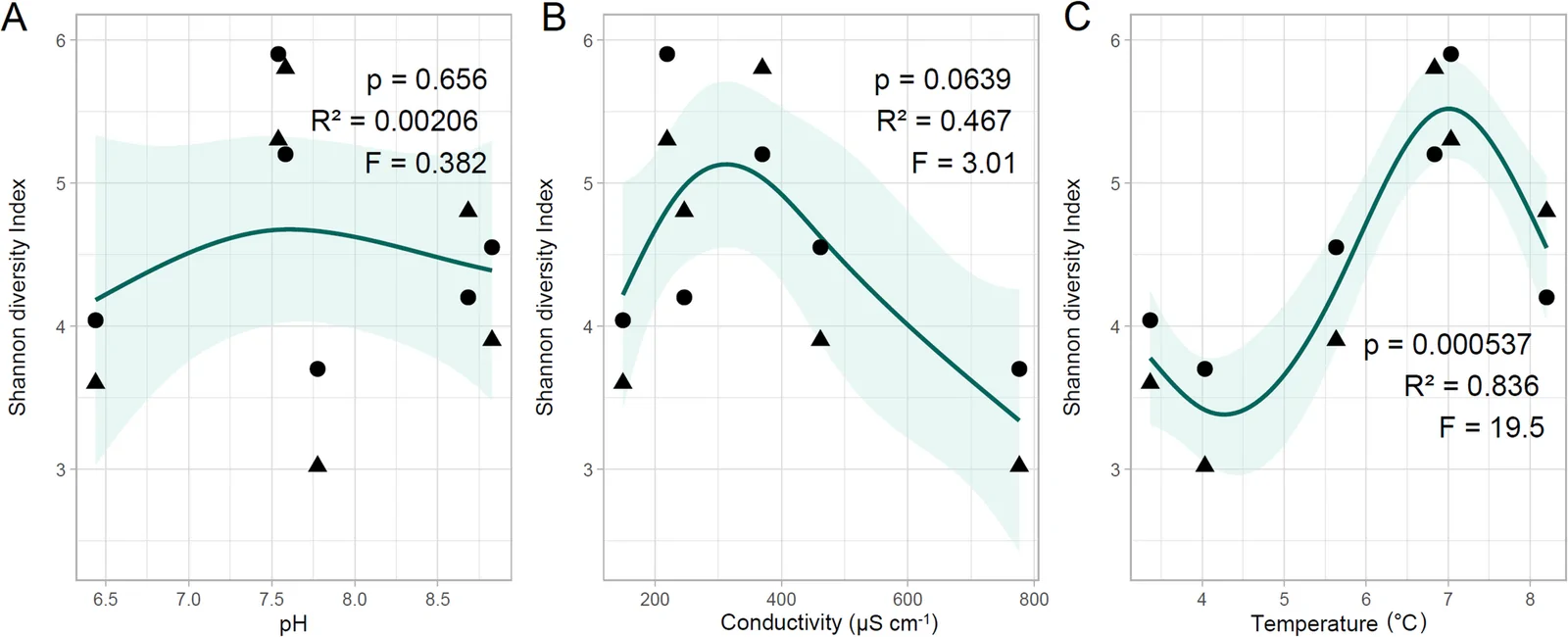
Associations between environmental variables and bacterial alpha diversity in the gut of *Branchinecta gaini*. (**A**–**C**) Generalized additive models (GAMs) describing associations between Shannon diversity index and measured physicochemical parameters of the water at the sampling sites: pH (**A**), conductivity (**B**), and temperature (**C**). Solid lines represent GAM smooth terms, and shaded areas indicate 95% confidence intervals. Statistical significance of the smooth terms is indicated on the plots. Circles represent gut content samples and triangles represent gut tract samples. Both sample types originate from the same pooled individuals and therefore represent paired, non-independent observations; therefore, these models are interpreted as exploratory. Given the limited number of observations and pooled sampling design, these relationships should be interpreted as exploratory and hypothesis-generating rather than confirmatory

Generalized additive models revealed contrasting relationships between environmental variables and bacterial alpha diversity in the gut, measured as the Shannon diversity index (Fig. 8). Shannon diversity showed no apparent relationship with pH (Fig. 8A), with the GAM explaining negligible variance (R² = 0.002, *p* = 0.656).

A non-linear but weak association was observed between conductivity and Shannon diversity (Fig. 8B). The GAM explained a moderate proportion of variance (R² = 0.467), although the smooth term was not statistically significant (*p* = 0.0639), indicating that this relationship should be interpreted cautiously.

In contrast, temperature showed a non-linear association with bacterial alpha diversity (Fig. 8C). Shannon diversity increased with temperature, and the GAM explained a large proportion of variance (R² = 0.836, F = 19.5, *p* = 0.000537). However, given the limited number of observations, this relationship should also be considered exploratory rather than confirmatory.

Together, these results indicate that environmental variables may be associated with different aspects of gut microbiome organization: conductivity with variation in community composition and temperature with within-sample diversity. These patterns should be interpreted as indicative and hypothesis-generating rather than definitive. Because gut content and gut tract samples were derived from the same pooled individuals, these data represent paired compartments rather than independent observations.

## Discussion

Our results suggest that feeding patterns and gut-associated bacterial communities of the Antarctic fairy shrimp *Branchinecta gaini* are closely associated with local environmental conditions rather than structured around a stable, host-specific intestinal microbiota. Across Antarctic freshwater ponds, *B. gaini* exhibits pronounced dietary flexibility, which is mirrored by highly variable bacterial communities in both gut contents and gut tract in pooled, pond-level samples. Despite consistent differentiation between gut microcompartments, taxonomic overlap of bacterial lineages among ponds remained low, suggesting that feeding ecology and environmental context may influence microbiota assembly more strongly than persistent host–microbe associations at the population level. Together, these findings are consistent with *B. gaini* as an ecologically flexible consumer whose intestinal microbiota largely reflects opportunistic feeding and site-specific environmental filtering [5, 21]. Below, we discuss how these patterns reflect the interplay between environmental filtering, gut microhabitats, and ecological plasticity in Antarctic freshwater systems.

### Dietary Composition and Feeding Behaviour

The diet of this Antarctic fairy shrimp varied considerably between sites. Microscopic analysis of gut contents revealed the presence of the following groups of organisms: diatoms, filamentous green algae, cyanobacteria, fungal hyphae and spores, rotifers, nematodes, tardigrades, and even fragments of bryophytes and vascular plants. The presence of *B. gaini* exoskeleton fragments and copepod eggs of the genus *Boeckella* suggests occasional cannibalism or predation on other zooplankton, as well as the consumption of shed exuviae. This high dietary diversity confirms the role of *B. gaini* as a feeding generalist in Antarctic water bodies, capable of utilizing both autochthonous production (algae and microorganisms developing within the pond) and allochthonous food sources (organic matter and organisms introduced by wind or marine animals). Consistent with Paggi [5] gut content analysis, we found that the diet of *B. gaini* is dominated by amorphous organic matter, benthic algae, and microbiological components such as fungal hyphae and spores. Importantly, we also frequently observed remnants of conspecific individuals in the gut contents, which supports Paggi [5] earlier hypothesis regarding scavenging behaviour. Hawes [14] provided direct behavioural evidence that *B. gaini* actively manipulates and consumes detrital material, including carrion from dead conspecifics. Our data corroborate this observation, documenting the ingestion of *B. gaini* remains under natural conditions.

Microscopic observations suggest that, apart from *B. gaini* remains, benthic diatoms and green algae are dominant dietary components. However, molecular analyses do not confirm this assumption, instead highlighting ciliates as the primary dietary element. It is likely that microscopy preferentially detects dietary elements with indigestible structures, such as cellulose cell walls or diatom frustules. Ciliate remnants, lacking such cell walls, were not identifiable under microscopic examination. The discrepancy between molecular and microscopic results underscores the limitations of microscopy-based dietary analyses and should be considered in future research of this kind. Nonetheless, both microscopic analysis and eukaryotic 18 S rRNA gene amplicon high-throughput sequencing reveal significant dietary variation across sampling sites. For example, sequences of *Oligohymenophorea* (ciliates) were abundant in specimens from Sphinx and Turret ponds but nearly absent in those from Ornithologists and Viéville. Similarly, green algae (e.g., *Trebouxiophyceae*) constituted a substantial portion of the gut content in Sphinx Pond (over 16% of sequences) but were negligible elsewhere. Differences in gut content composition of *B. gaini* among ponds can be plausibly explained by pronounced spatial heterogeneity of Antarctic freshwater ecosystems [35]. Previous studies have shown that microbial and algal communities in Antarctic lakes and ponds vary strongly in response to local physicochemical conditions, resulting in distinct site-specific assemblages [36, 37]. These observations suggest that each pond or lake offers *B. gaini* a specific food base shaped by local environmental conditions and the influx of nutrients from terrestrial and marine ecosystems. Ponds with greater nutrient input may develop richer algal blooms and microbial mats, which *B. gaini* readily exploits. For example, the proximity of seabird colonies can substantially enrich water bodies with nitrogen and phosphorus from guano, promoting algal growth [38]. Indeed, some of our sites (e.g., Rakusa Pond) are located near gentoo penguin colonies. However, we did not observe a straightforward correlation between this factor and algal abundance in the diet, although at this site elevated contribution of blue-green algae in gut contents was noted. This is consistent with published observation of nitrophilic cyanobacteria developing in freshwater ponds adjacent to marine birds and marine mammals breeding/resting sites [39]. At the Ornithologists Pond, situated on a hill with minimal avian influence, green algae were significantly more prominent in the diet, confirming their preference for oligotrophic freshwaters [38]. Interestingly, in the Ecology Pond, where algal blooms were visually apparent as green turbidity, algae were not the dominant dietary component and were surpassed by ciliates. The high relative abundance of *Oligohymenophorea* detected in gut contents likely reflects a combination of biological and methodological factors. Members of this group are common components of Antarctic freshwater biofilms, periphyton, and benthic microbial mats, and many species occur as epibionts on invertebrates or organic substrates [40]. Given the detritus-oriented feeding behaviour of *B. gaini* and its frequent interaction with benthic material, biofilms, and conspecific remains, ingestion of epibiotic and surface-associated ciliates is biologically plausible. In addition, consumption of exuviae or dead individuals, which provide suitable substrates for ciliate colonization, may further increase exposure to *Oligohymenophorea*. At the same time, methodological biases associated with 18 S rRNA gene metabarcoding should be considered, as ciliates are characterized by high rRNA gene copy numbers and are efficiently amplified by commonly used primer sets [41].

The presence of moss and grass fragments in the gut contents from some sites suggests inputs of terrestrial material: ponds surrounded by dense moss carpets or clumps of Antarctic hair grass (*D. antarctica*) likely provide plant detritus. *B. gaini* feeding in such ponds consumes decaying moss fragments, along with the fungi and microorganisms inhabiting this detritus. These moss-dominated systems may support a distinct trophic base (e.g., detritivorous organisms associated with fungi and terrestrial algae), often substantially adding to the dietary profile of *B*. *gaini* in certain sites. Additionally, sporadic ingestion of avian-derived material was recorded—we detected small feather fragments in gut contents from Ecology and especially from the Rakusa site.

Although *B. gaini* is not a specialist bacterivore, bacteria likely represent an unavoidable and potentially important dietary component, ingested together with detritus, biofilms, and microalgae during feeding. Similar detritus-based bacterial ingestion has been reported for other freshwater crustaceans, where bacteria enhance the nutritional quality of ingested material rather than serving as a primary food source [42]. Bacterial biomass contributes proteins, lipids, and essential micronutrients when ingested together with detrital and biofilm-associated material [43]. This interpretation is supported by aquaculture studies on anostracans, where the presence of bacterial communities in rearing systems has been shown to enhance growth, survival, and nutritional quality of cultured individuals, even when bacteria are not provided as a targeted food source [44]. Aquaculture studies on filter-feeding anostracans such as *Artemia* suggest that bacteria can contribute to nutrition when ingested, either directly or as part of mixed microbial aggregates, and in some experimental diets have supported growth and biomass comparable to algal feeds [45].

Another component frequently observed in the gut contents was inorganic mineral particles (e.g., fine sand and silt), present in all examined individuals, often in substantial quantities. These particles are likely ingested unintentionally during benthic foraging or while filtering turbid water, including wind-blown dust mixed with organic detritus.

Mineral particles may influence digestive processes both physically and chemically. In crustaceans, clays have been shown to bind toxins and modulate gut microbiota, while in branchiopods they may enhance digestibility by mechanically disrupting resilient food structures [46–48]. This mechanical role is analogous to gastroliths in granivorous birds. Together, these observations suggest that ingested mineral particles may contribute to digestion through a combination of physicochemical and mechanical effects.

Among the non-dietary components detected in the gut contents of *B. gaini*, microplastics were particularly notable. Synthetic fibres were observed during dissection, including red fibres (approximately 2–5 mm long) in three individuals from the Llano site and a blue fibre fragment in one specimen from Viéville. In Llano individuals, small red fragments suggested partial shredding of fibres by the mandibles, whereas the digestive tract of the Viéville specimen was nearly empty, potentially indicating difficulty in processing the ingested plastic and interference with normal food intake. Based on their morphology, the fibres were identified as plastics, most likely polyester. Although alarming, the presence of microplastics in Antarctic freshwater invertebrates is increasingly reported and reflects growing environmental contamination in the region [49–51]. Importantly, experimental studies have demonstrated that *B*. *gaini* exhibits physiological and molecular responses to plastic exposure, even in the absence of significant mortality. Exposure to polystyrene nanoparticles has been shown to induce histopathological changes in the gut epithelium, altered ventilation rates, and upregulation of stress-related genes, alongside sublethal effects such as increased moulting frequency and disrupted faecal structure [18]. Together, these findings suggest that microplastic ingestion may have chronic consequences for digestion, energy balance, and nutrient cycling in Antarctic anostracans.

The combined use of microscopy and metabarcoding provides complementary insights into the feeding ecology of *B*. *gaini* and the structure of gut-associated communities. Microscopy captures morphologically identifiable and structurally persistent dietary components, such as diatom frustules, plant fragments, and metazoan remains, thereby providing information on ingested material that resists digestion. In contrast, metabarcoding detects a broader spectrum of taxa, including soft-bodied and rapidly degraded organisms such as ciliates and other protists, as well as microbial components that are not identifiable microscopically.

Importantly, the integration of these approaches allows differentiation between structurally preserved dietary signals and more transient or molecularly detectable components, highlighting potential biases inherent to each method when used in isolation. The observed discrepancies between microscopy and sequencing therefore do not represent inconsistencies but rather reflect complementary aspects of feeding and gut-associated assemblages. Together, these methods provide a more comprehensive view of trophic interactions and microbiome structure than either approach alone.

### Gut Tract as a Weak Ecological Filter Rather Than a Passive Conduit

A central question in gut microbiome ecology is whether microbial communities detected in animal intestines primarily reflect passive transit of ingested material or are shaped by selective processes within the gut environment [52, 53]. In aquatic invertebrates, both scenarios have been reported, ranging from largely transient microbiota closely tracking surrounding environmental communities to systems characterized by stable, host-associated core assemblages [17]. In our pooled, pond-level samples, bacterial communities differed between gut contents and gut tract, and Bray–Curtis dissimilarities between these compartments remained high even within ponds. These patterns are not consistent with a purely passive transit model, although they do not allow inference at the level of individual hosts.

At the same time, gut tract–associated bacterial communities did not converge across sites, and no stable core microbiota was detected at the composite sample scale, contrasting with patterns observed in organisms exhibiting strong host control over microbial assembly, such as insects with vertically transmitted symbionts or vertebrates [54]. Instead, gut tract communities retained pronounced pond-specific signatures, indicating that environmental source pools exert a dominant influence on microbiota composition.

This pattern is consistent with recent work on *B. gaini* showing that intestinal microbiomes are shaped by variable, rather than homogenizing, selection processes. Such site-dependent selection allows host filtering to operate on locally available environmental source pools, resulting in distinct gut microbiota across ponds rather than convergence toward a single host-specific assemblage [21]. In this framework, host-related filtering and environmental sourcing are not mutually exclusive but operate jointly, with local environmental context determining the pool of bacteria upon which gut-level selection can act. Differential association of bacteria with the gut tract may reflect a combination of passive retention on the peritrophic matrix, bacterial cell surface properties, and host-mediated processes. Because our data do not resolve fine-scale spatial variation within the gut, the relative contribution of these mechanisms cannot be determined. Although direct evidence for differential bacterial adhesion to the peritrophic matrix in anostracans is limited, studies of gut microbiome assembly in related crustaceans suggest that the peritrophic matrix acts as a semi-permeable physical filter that constrains microbial colonization and favours the transient passage of ingested material [16]. In arthropods, peritrophic matrix structures are known to limit microbe–epithelium interactions, and by analogy, only bacterial taxa with suitable surface properties or those associated with particle-bound biofilms may be retained longer within the gut environment, while others are rapidly cleared [55]. Consistent with this interpretation, gut tract–associated communities in *B. gaini* were enriched in bacterial families characterized by particle-associated or biofilm-forming life strategies, including *Comamonadaceae*, *Rhodobacteraceae*, and *Verrucomicrobiaceae*. Members of these groups are widely recognized as dominant constituents of aquatic biofilms and particle-associated communities, where surface attachment confers stability and persistence under flow or gut passage conditions (e.g., *Comamonadaceae* in freshwater biofilms [56]; *Rhodobacteraceae* and close relatives in surface-associated communities [57]; *Verrucomicrobiaceae* in particulate biofilms [58]; biofilm ecology fundamentals [59]). The apparent persistence of these taxa likely reflects a combination of passive retention on the peritrophic matrix, tolerance to gut physicochemical conditions, and association with ingested particles or biofilms, rather than stable colonization.

Variation in gut tract bacterial diversity among ponds further suggests a possible role of environmental context. However, observed differences in alpha diversity should be interpreted cautiously, as they depend on sequencing depth normalization and the compositional nature of amplicon data. Nonetheless, higher gut tract diversity likely reflects a richer and more diverse environmental source pool, rather than prolonged host–microbe coevolution. Alternatively, elevated diversity in some ponds may be linked to greater ecological stability, allowing repeated exposure of *B. gaini* populations to similar microbial assemblages over time; however, disentangling such historical effects from contemporary environmental filtering would require temporal or experimental data. In this context, differences among ponds may also reflect contrasting developmental histories. Turret, Sphinx, and Ornithologists represent relatively long-established freshwater systems, whereas Llano, Ecology, and Viéville formed more recently following glacier retreat [11, 23, 60, 61]. Such differences in system age may influence the complexity and stability of microbial and microeukaryotic source pools, thereby contributing to the observed variation in gut content composition and gut tract bacterial diversity [36, 62].

Together, these patterns support a model in which the gut tract of *B. gaini* functions as a weak ecological filter rather than a tightly regulated microbial niche. Similar environmentally acquired and weakly selected gut microbiomes have been reported for other freshwater invertebrates and zooplankton, particularly those with opportunistic feeding strategies and frequent contact with detrital and biofilm-associated microbial communities. For example, the gut microbiome of freshwater zooplankton has been shown to be highly flexible and strongly influenced by external environmental conditions with little evidence of a stable core community, indicating a dominant role of environmental sourcing over strict host control [63]. Additionally, studies on zooplankton-associated bacteria suggest that microbial assemblages are closely linked to environmental parameters such as dissolved organic carbon and nutrient status, reflecting a transient interaction with the host rather than stable colonization [64], and zooplankton can act as temporary reservoirs for environmentally abundant bacterial taxa [65] — patterns that are consistent with weak ecological filtering of gut microbiota in opportunistic aquatic consumers. Given that the peritrophic matrix is continuously shed, association with the gut tract fraction does not necessarily imply stable host association. Accordingly, bacteria associated with the gut tract fraction should not be interpreted as strictly host-associated, but rather as representing microbes retained within gut-associated structures, including the peritrophic matrix. The observed patterns are consistent with differential retention or clearance processes, although the underlying mechanisms cannot be resolved with the present data.

### Hydrological Connectivity and Temperature as Distinct Drivers of Gut Microbiome Composition and Diversity

Observed relationships between environmental parameters and gut-associated bacterial communities of *B. gaini* likely reflect differences in hydrological connectivity and physicochemical stability among the studied postglacial lakes. However, these findings should be interpreted as exploratory, given the limited number of sites and lack of replication.

Although such lakes are generally expected to exhibit low conductivity, Llano and Ecology are situated at lower elevations and are periodically influenced by marine water intrusions, followed by dilution through rainfall and snowmelt. In contrast, Viéville Lake is characterized by a continuous input of glacial meltwater and limited marine influence, resulting in lower and more stable conductivity [66]. Previous work has shown that *B. gaini* itself is tolerant to salinity fluctuations within this range [10], suggesting that the significant association observed here between conductivity and gut bacterial community composition does not reflect direct physiological stress on the host. Instead, conductivity likely acts indirectly by reshaping the surrounding microbial pool, either through the introduction of marine or estuarine bacteria or through shifts in the relative abundances of freshwater taxa. Salinity gradients and hydrological connectivity have been shown to act as strong predictors of microbial community composition across polar and coastal aquatic environments, often outweighing temperature or pH effects [67, 68].

In contrast to conductivity, temperature was not significantly associated with overall gut community composition but showed a significant non-linear relationship with bacterial alpha diversity. Lakes that are isolated from both marine intrusions and glacial runoff tended to exhibit higher water temperatures, likely due to the absence of cold-water inputs. Elevated temperatures may enhance bacterial metabolic activity and increase the number of ecological niches available within the gut environment, thereby supporting higher within-sample diversity without necessarily favouring specific bacterial taxa. This decoupling between drivers of community composition and diversity suggests that distinct ecological processes operate at different organizational levels of the gut microbiome, with temperature regulating local carrying capacity rather than taxonomic identity. Such effects may be particularly pronounced in polar environments, where relatively small temperature differences can have disproportionate biological consequences. Non-polar region studies in aquatic ecosystems have shown that water temperature can be a dominant driver of bacterial community diversity, sometimes outweighing other environmental predictors (e.g., temperature gradients shaped bacterial diversity patterns more strongly than other variables in lakes across the Qinghai-Tibetan and Inner Mongolia Plateaus) and warming scenarios have been demonstrated to significantly modify bacterial community assembly and diversity in freshwater mesocosms and seasonal time series experiments [69, 70]. Moreover, in riverine systems increases in temperature were associated with higher planktonic bacterial diversity, highlighting a broader pattern linking temperature changes with bacterial alpha diversity in aquatic habitats [71].

Water pH showed no detectable relationship with either gut bacterial community composition or diversity. This lack of association likely reflects the multifactorial controls on pH in these systems, including marine water inputs, primary production, and nitrogen cycling processes such as ammonification, which may obscure any direct linkage with microbial assemblages [72–74]. Additionally, gut conditions may be partially buffered from external pH variability by host-mediated processes [75]. Together, these results indicate that environmental drivers may influence gut-associated bacterial communities of *B. gaini* through multiple, partially independent pathways, with hydrological connectivity shaping community composition *via* the regional microbial pool and temperature modulating within-gut diversity. These interpretations remain tentative, and the observed relationships should be viewed as hypothesis-generating rather than confirmatory. Because gut content and gut tract samples were derived from the same pooled individuals, they cannot be considered independent replicates. Accordingly, differences between compartments should be interpreted as paired, within-site contrasts rather than independent patterns.

### Limitations and Directions for Future Research

Several limitations of this study should be acknowledged when interpreting the observed patterns in diet composition and gut-associated microbial communities of *B. gaini*. First, due to the low biomass of individual specimens, individuals were pooled prior to DNA extraction and sequencing, resulting in composite samples representing each site. Consequently, within-site biological variability could not be assessed, and statistical inference is limited to descriptive, site-level patterns rather than individual-level variation. We acknowledge that pooling may obscure individual-level variation and potentially amplify the influence of atypical individuals. Pooling may also affect the detection of rare ASVs and influence richness estimates, particularly for alpha diversity metrics. Nevertheless, composite sampling represents a pragmatic and commonly applied approach in Antarctic freshwater systems, and the consistent detection of dominant taxa across sites supports the robustness of the broad-scale patterns reported here [76]. Furthermore, the findings of Schwob and colleagues [21] indicate that the gut bacteriome of Antarctic *B*. *gaini* was surprisingly homogeneous within individuals from the same site in terms of diversity and community structure, hinting that within-site sample pooling may introduce minimal bias in a site to site comparison model.

Second, the limited number of sampled ponds (*n* = 8) constrains the statistical power to detect and generalize relationships between environmental variables and microbial diversity. Although generalized additive models revealed strong and significant associations between temperature and bacterial alpha diversity, and conductivity with community composition, these relationships should be interpreted cautiously and primarily as exploratory. Future studies incorporating a larger number of sites and repeated temporal sampling would allow more robust testing of environmental drivers and improve inference regarding causality.

Third, amplicon-based metabarcoding approaches introduce several well-recognized biases. Primer choice, differential amplification efficiency, and variation in rRNA gene copy numbers can substantially influence the observed taxonomic composition of microbial communities [77–79]. These limitations are particularly relevant in Antarctic environments, where many microbial taxa remain poorly represented in reference databases [35, 62]. In addition, sequence-based microbiome data are inherently compositional and do not provide information on absolute abundances, which constrains quantitative interpretation of relative changes among taxa [80, 81]. Consequently, estimates of alpha diversity and relative taxonomic composition should be interpreted as method-dependent and conservative, particularly with respect to the magnitude of observed differences.

Relatedly, distinguishing between ingested dietary items and putative resident gut-associated microorganisms remains challenging using amplicon data alone. Bacterial and eukaryotic taxa detected in gut samples may represent transient dietary components, environmentally acquired microbes passing through the gut, or taxa capable of short-term persistence or proliferation within the gut environment. While the comparison of gut content and gut tract–associated fractions provides partial insight into this distinction, definitive discrimination between transient and resident microbes would require complementary approaches, such as comparisons with environmental (water and sediment) microbial communities, starvation or clearance experiments, or microscopy-based localization of microbes within gut tissues. These integrative strategies have been emphasized in host–microbiome research to separate environmentally acquired, transient microbes from those persistently associated with host gut environments and active in host functions [82, 83]. Because our study relied exclusively on amplicon sequencing, we could not determine the physical location of bacteria within the gut environment. Consequently, taxa commonly associated with biofilms may have originated from ingested particle-associated biofilms, biofilms developing on gut-associated surfaces, or free-living cells, and these alternatives cannot be distinguished using the present dataset. Despite these limitations, the integration of microscopy with 18 S and 16 S rRNA gene metabarcoding across multiple Antarctic ponds provides a comprehensive and complementary view of *B. gaini* feeding ecology and gut-associated microbial variability. Future studies combining higher-resolution temporal sampling, environmental microbial baselines, and experimental manipulations will be essential to further disentangle the relative roles of diet, environment, and host filtering in shaping gut microbiota in polar freshwater invertebrates. These findings should be interpreted within the context of Antarctic freshwater systems, where comparable data remain scarce, and caution is warranted when extrapolating to other taxa or ecosystems.

### Conclusions

Our results suggest that the feeding ecology and gut-associated bacterial communities of the Antarctic fairy shrimp *B*. *gaini* are closely associated with local environmental conditions at the pooled, pond-level scale. Across postglacial freshwater ponds, pronounced dietary flexibility is mirrored by highly variable and site-specific gut bacterial assemblages, characterized by low taxonomic overlap among ponds and the absence of a consistent core microbiota. Although bacterial communities differed between gut contents and gut tract, patterns are consistent with limited host-related filtering rather than strong host control, and gut-associated assemblages largely reflected environmentally acquired source pools. Associations between hydrological connectivity and conductivity gradients were observed in relation to shifts in bacterial community composition, while temperature showed a significant association with bacterial alpha diversity, however these relationships should be interpreted as exploratory. Together, these findings are consistent with *B*. *gaini* functioning as an ecologically flexible consumer whose gut microbiome reflects opportunistic feeding and environmental context.

## Supplementary Information

Below is the link to the electronic supplementary material.

Supplementary Material 1

## Data Availability

Raw sequencing data have been deposited in the NCBI Sequence Read Archive (SRA) under the BioProject accession numbers PRJNA1315029 (bacterial amplicons) and PRJNA1321750 (eukaryotic amplicons).

## References

[1] Dartnall H (2017) The freshwater fauna of the South Polar Region: A 140-year review. Pap Proc R Soc Tasman 151:19–57. 10.26749/rstpp.151.19

[2] Maturana CS, Bernal-Durán V, Gañán M et al (2025) Bridging the Scotia Arc: Climate-Driven Shifts in Connectivity of the Freshwater Crustacean Branchinecta gaini in Sub-Antarctic and Antarctic Ecosystems. Divers Distrib 31:e70049. 10.1111/ddi.70049

[3] Peck LS (2004) Physiological flexibility: the key to success and survival for Antarctic fairy shrimps in highly fluctuating extreme environments. Freshw Biol 49:1195–1205. 10.1111/j.1365-2427.2004.01264.x

[4] Pokorný M, Cohen RG, Nedbalová L et al (2024) South! Phylogeography of the Antarctic fairy shrimp Branchinecta gaini and its closest Patagonian congener Branchinecta granulosa reveals a long-term association of freshwater fauna with the southern continent. Org Divers Evol. 10.1007/s13127-024-00654-x

[5] Paggi JC (1996) Feeding ecology of Branchinecta gaini (Crustacea: Anostraca) in ponds of South Shetland Islands, Antarctica. Polar Biol 16:13–18

[6] Rochera C, Camacho A (2019) Limnology and Aquatic Microbial Ecology of Byers Peninsula: A Main Freshwater Biodiversity Hotspot in Maritime Antarctica. Diversity 11:201. 10.3390/d11100201

[7] Ellis-Evans JC (1996) Microbial diversity and function in Antarctic freshwater ecosystems. Biodivers Conserv 5:1395–1431. 10.1007/BF00051985

[8] Pertierra LR, Convey P, Barbosa A et al (2025) Advances and shortfalls in knowledge of Antarctic terrestrial and freshwater biodiversity. Science 387:609–615. 10.1126/science.adk2118 39913585

[9] Hawes TC, Worland MR, Bale JS (2008) Physiological constraints on the life cycle and distribution of the Antarctic fairy shrimp Branchinecta gaini. Polar Biol 31:1531–1538. 10.1007/s00300-008-0493-1

[10] Cukier S, Grzesiak J, Bialik RJ (2025) Ecological and Genetic Insights Into Antarctic Fairy Shrimp, Branchinecta gaini Daday, 1910 (Branchiopoda: Anostraca) Populations on King George Island, Antarctica. Freshw Biol 70:e70144. 10.1111/fwb.70144

[11] Jurasz W, Kittel W, Presler P (1983) Life cycle of Branchinecta gaini Daday, 1910, (Branchiopoda, Anostraca) from King George Island, South Shetland Islands*). Pol Polar Res 1:143–154

[12] Brendonck L, Rogers DC, Olesen J et al (2008) Global diversity of large branchiopods (Crustacea: Branchiopoda) in freshwater. Hydrobiologia 595:167–176. 10.1007/s10750-007-9119-9

[13] Chaoruangrit L, Plodsomboon S, Rogers DC, Sanoamuang L (2017) Morphology of mandibles and food size in two fairy shrimps (Branchiopoda: Anostraca) from Thailand. J Crustac Biol 37:579–587. 10.1093/jcbiol/rux058

[14] Hawes TC (2008) Feeding behaviour in the Antarctic fairy shrimp, Branchinecta gaini. Polar Biol 31:1287–1289. 10.1007/s00300-008-0494-0

[15] Ampe F, Thiéry A (1998) Microflora associated with the digestive tract of the fairy shrimp Branchinella spinosa (H. Milne Edwards, 1840) (Crustacea, Branchiopoda). FEMS Microbiol Lett 158:201–205. 10.1111/j.1574-6968.1998.tb12821.x

[16] Martin GG, Natha Z, Henderson N et al (2020) Absence of a microbiome in the midgut trunk of six representative Crustacea. J Crustac Biol 40:122–130. 10.1093/jcbiol/ruz087

[17] Ganesan R, Wierz JC, Kaltenpoth M, Flórez LV (2022) How It All Begins: Bacterial Factors Mediating the Colonization of Invertebrate Hosts by Beneficial Symbionts. Microbiol Mol Biol Rev 86:e00126–e00121. 10.1128/mmbr.00126-21 PMC9769632 36301103

[18] Bergami E, Krupinski Emerenciano A, Palmeira Pinto L et al (2022) Behavioural, physiological and molecular responses of the Antarctic fairy shrimp Branchinecta gaini (Daday, 1910) to polystyrene nanoplastics. NanoImpact 28:100437. 10.1016/j.impact.2022.100437 36332901

[19] Pociecha A (2007) Effect of temperature on the respiration of an Antarctic freshwater anostracan, Branchinecta gaini Daday 1910, in field experiments. Polar Biol 30:731–734. 10.1007/s00300-006-0230-6

[20] Ralph R (1967) The osmotic and ionic regulation of *Branchinecta gaini* Daday. Philos Trans R Soc Lond B Biol Sci 252:339–341. 10.1098/rstb.1967.0022

[21] Schwob G, Cabrol L, Vidal P et al (2024) Which microbiome are we talking about? Contrasted diversity patterns and eco-evolutionary processes between gill and intestinal microbiomes of Antarctic fairy shrimps. Front Ecol Evol 12. 10.3389/fevo.2024.1438057

[22] Fabiszewski J, Wojtuń B (1997) The occurrence and development of peat mounds on King George Island (Maritime Antarctic)

[23] Pudełko R (2003) Topographic map of the SSSI 8, King George Island, West Antarctica. Pol Polar Res 24:53–60

[24] R Core Team (2022) R: A language and environment for statistical computing

[25] Znój A, Grzesiak J, Gawor J et al (2022) Highly specialized bacterial communities within three distinct rhizocompartments of Antarctic hairgrass (Deschampsia antarctica Desv). Polar Biol 45:1–12. 10.1007/s00300-022-03027-2

[26] Damborenea C, Rogers DC, Thorp JH (2020) Thorp and Covich’s freshwater invertebrates, 4th edn. Academic press, an imprint of Elsevier, Amsterdam

[27] Dory F, Nava V, Nedbalovà L et al (2025) Morphological diversity of microalgae and Cyanobacteria of cryoconite holes in Northern Victoria Land, Antarctica. J Glaciol 71:e37. 10.1017/jog.2025.12

[28] Sabbe K, Verleyen E, Hodgson DA et al (2003) Benthic diatom flora of freshwater and saline lakes in the Larsemann Hills and Rauer Islands, East Antarctica. Antarct Sci 15:227–248. 10.1017/S095410200300124X

[29] Janiec K (1996) The comparison of freshwater invertebrates of Spitsbergen (Arctic) and King George Island (Antarctic). Pol POLAR Res 17:173–202

[30] Klindworth A, Pruesse E, Schweer T et al (2013) Evaluation of general 16S ribosomal RNA gene PCR primers for classical and next-generation sequencing-based diversity studies. Nucleic Acids Res 41:e1. 10.1093/nar/gks808 PMC3592464 22933715

[31] (2026) OpenGene/fastp

[32] Andrews S (2010) Babraham bioinformatics - FastQC a quality control tool for high throughput sequence data. https://www.bioinformatics.babraham.ac.uk/projects/fastqc/. Accessed 26 Sept 2025

[33] Bolyen E, Rideout JR, Dillon MR et al (2019) Reproducible, interactive, scalable and extensible microbiome data science using QIIME 2. Nat Biotechnol 37:852–857. 10.1038/s41587-019-0209-9 PMC7015180 31341288

[34] Callahan B (2024) Silva taxonomic training data formatted for DADA2 (Silva version 138.2) [Data set]. Zenodo. 10.5281/zenodo.14169026

[35] Cavicchioli R (2015) Microbial ecology of Antarctic aquatic systems. Nat Rev Microbiol 13:691–706. 10.1038/nrmicro3549 26456925

[36] Kollár J, Kopalová K, Kavan J et al (2023) Recently formed Antarctic lakes host less diverse benthic bacterial and diatom communities than their older counterparts. FEMS Microbiol Ecol 99:fiad087. 10.1093/femsec/fiad087 PMC10446143 37516444

[37] Logares R, Lindström E, Langenheder S et al (2012) Biogeography of bacterial communities exposed to progressive long-term environmental change. ISME J. 10.1038/ismej.2012.168. 7:PMC3635229 23254515

[38] Otero XL, De Peña-Lastra L, Pérez-Alberti S A, et al (2018) Seabird colonies as important global drivers in the nitrogen and phosphorus cycles. Nat Commun 9:246. 10.1038/s41467-017-02446-8 PMC5780392 29362437

[39] Zębek E, Napiórkowska-Krzebietke A, Świątecki A, Górniak D (2021) Biodiversity of periphytic cyanobacteria and algae assemblages in polar region: a case study of the vicinity of Arctowski Polish Antarctic Station (King George Island, Antarctica). Biodivers Conserv 30. 10.1007/s10531-021-02219-2

[40] Jung J-H, Park K-M, Yang EJ et al (2015) Patchy-distributed ciliate (Protozoa) diversity of eight polar communities as determined by 454 amplicon pyrosequencing. Anim Cells Syst 19:339–349. 10.1080/19768354.2015.1082931

[41] Gong J, Dong J, Liu X, Massana R (2013) Extremely High Copy Numbers and Polymorphisms of the rDNA Operon Estimated from Single Cell Analysis of Oligotrich and Peritrich Ciliates. Protist 164:369–379. 10.1016/j.protis.2012.11.006 23352655

[42] Anderson TR, Pond DW, Mayor DJ (2017) The role of microbes in the nutrition of detritivorous invertebrates: a stoichiometric analysis. Front Microbiol 7. 10.3389/fmicb.2016.02113 PMC5209341 28101083

[43] Fernandes da Silva C, Ballester E, Monserrat J et al (2008) Contribution of microorganisms to the biofilm nutritional quality: protein and lipid contents. Aquac Nutr 14:507–514. 10.1111/j.1365-2095.2007.00556.x

[44] Toi HT, Boeckx P, Sorgeloos P et al (2013) Bacteria contribute to *Artemia* nutrition in algae-limited conditions: A laboratory study. Aquaculture 388–391:1–7. 10.1016/j.aquaculture.2013.01.005

[45] Ogello EO, Outa NO, Mukaburu BO, Muthoka M (2023) Biofloc and green water condition improves reproductive traits and fatty acid composition of Artemia franciscana cultured under limited algal conditions. Aquac Fish Fish 3:61–70. 10.1002/aff2.89

[46] Ng W-K, Mong M-L, Abdul-Hamid A-A (2023) Dietary montmorillonite clay improved *Penaeus vannamei* survival from acute hepatopancreatic necrosis disease and modulated stomach microbiota. Anim Feed Sci Technol 297:115581. 10.1016/j.anifeedsci.2023.115581

[47] Damato A, Vianello F, Novelli E et al (2022) Comprehensive Review on the Interactions of Clay Minerals With Animal Physiology and Production. Front Vet Sci 9:889612. 10.3389/fvets.2022.889612 PMC9127995 35619608

[48] Maeda-Martínez AM, Obregón-Barboza H, Dumont HJ (1995) Laboratory culture of fairy shrimps using baker’s yeast as basic food in a flow-through system. In: Belk D, Dumont HJ, Maier G (eds) Studies on Large Branchiopod Biology and Aquaculture II. Springer Netherlands, Dordrecht, pp 141–157

[49] González-Pleiter M, Edo C, Velázquez D et al (2020) First detection of microplastics in the freshwater of an Antarctic Specially Protected Area. Mar Pollut Bull 161:111811. 10.1016/j.marpolbul.2020.111811 33157507

[50] Gurumoorthi K, Luis AJ (2023) Recent trends on microplastics abundance and risk assessment in coastal Antarctica: Regional meta-analysis. Environ Pollut 324:121385. 10.1016/j.envpol.2023.121385 36868550

[51] Pellegrino D, La Russa D, Barberio L (2025) Pollution Has No Borders: Microplastics in Antarctica. Environments 12:77. 10.3390/environments12030077

[52] Flores C, Millard S, Seekatz AM (2025) Bridging Ecology and Microbiomes: Applying Ecological Theories in Host-associated Microbial Ecosystems. Curr Clin Microbiol Rep 12:9. 10.1007/s40588-025-00246-z PMC12000275 40248762

[53] Unzueta-Martínez A, Welch H, Bowen JL (2022) Determining the Composition of Resident and Transient Members of the Oyster Microbiome. Front Microbiol 12:828692. 10.3389/fmicb.2021.828692 PMC8847785 35185836

[54] Wilde J, Slack E, Foster KR (2024) Host control of the microbiome: Mechanisms, evolution, and disease. Science 385:eadi3338. 10.1126/science.adi3338 39024451

[55] Rodgers FH, Gendrin M, Wyer CAS, Christophides GK (2017) Microbiota-induced peritrophic matrix regulates midgut homeostasis and prevents systemic infection of malaria vector mosquitoes. PLOS Pathog 13:e1006391. 10.1371/journal.ppat.1006391 PMC5448818 28545061

[56] Willems A (2014) The family Comamonadaceae. The Prokaryotes: Alphaproteobacteria and Betaproteobacteria. Springer, Berlin Heidelberg, pp 777–851. https://link.springer.com/rwe/10.1007/978-3-642-30197-1_238

[57] Luo H, Moran MA (2014) Evolutionary Ecology of the Marine Roseobacter Clade. Microbiol Mol Biol Rev 78:573–587. 10.1128/mmbr.00020-14 PMC4248658 25428935

[58] Sangwan P, Kovac S, Davis KER et al (2005) Detection and Cultivation of Soil Verrucomicrobia. Appl Environ Microbiol 71:8402–8410. 10.1128/AEM.71.12.8402-8410.2005 PMC1317444 16332828

[59] Flemming H-C, Wuertz S (2019) Bacteria and archaea on Earth and their abundance in biofilms. Nat Rev Microbiol 17:247–260. 10.1038/s41579-019-0158-9 30760902

[60] Paulo A, Tokarski A (1982) Geology of turret point, three sisters point area, King George Island (South Shetland Islands, Antarctica). Stud Geol Pol 74:81–103

[61] Rosa KK da, Oliveira MAG de, Petsch C et al (2022) Expansion of glacial lakes on Nelson and King George Islands, Maritime Antarctica, from 1986 to 2020. Geocarto Int 37:4454–4464. 10.1080/10106049.2021.1886342

[62] Wilkins D, Yau S, Williams TJ et al (2013) Key microbial drivers in Antarctic aquatic environments. FEMS Microbiol Rev 37:303–335. 10.1111/1574-6976.12007 23062173

[63] Eckert EM, Anicic N, Fontaneto D (2021) Freshwater zooplankton microbiome composition is highly flexible and strongly influenced by the environment. Mol Ecol 30:1545–1558. 10.1111/mec.15815 33484584

[64] Wang Q, Hao Z, Ding R et al (2021) Host dependence of zooplankton-associated microbes and their ecological implications in freshwater Lakes. Water 13. 10.3390/w13212949

[65] Di Cesare A, Riva F, Colinas N et al (2022) Zooplankton as a Transitional Host for Escherichia coli in Freshwater. Appl Environ Microbiol 88:e02522–e02521. 10.1128/aem.02522-21 PMC9088391 35416683

[66] Nędzarek A, Pociecha A (2010) Limnological characterization of freshwater systems of the Thomas Point Oasis (Admiralty Bay, King George Island, West Antarctica). Polar Sci 4:457–467. 10.1016/j.polar.2010.05.008

[67] George SF, Fierer N, Levy JS, Adams B (2021) Antarctic water tracks: microbial community responses to variation in soil moisture, pH, and salinity. Front Microbiol 12. 10.3389/fmicb.2021.616730 PMC7873294 33584618

[68] Lew S, Glińska-Lewczuk K, Burandt P et al (2022) Salinity as a determinant structuring microbial communities in Coastal Lakes. Int J Environ Res Public Health 19. 10.3390/ijerph19084592 PMC9028135 35457457

[69] Liu X, Pan B, Wang L et al (2024) Water temperature and salt ions respectively drive the community assembly of bacterial generalists and specialists in diverse plateau lakes. Sci Total Environ 949:175271. 10.1016/j.scitotenv.2024.175271 39102958

[70] Yang Q, Yan Y, Huang J et al (2023) The impact of warming on assembly processes and diversity patterns of bacterial communities in mesocosms. Microorganisms 11. 10.3390/microorganisms11112807 PMC10672829 38004818

[71] Liu N, Wang B, Yang M et al (2023) The different responses of planktonic bacteria and archaea to water temperature maintain the stability of their community diversity in dammed rivers. Ecol Process 12:25. 10.1186/s13717-023-00438-9

[72] Hamdan M, Byström P, Hotchkiss ER et al (2018) Carbon dioxide stimulates lake primary production. Sci Rep 8:10878. 10.1038/s41598-018-29166-3 PMC6052161 30022034

[73] Jin Q, Kirk MF (2018) pH as a primary control in environmental microbiology: 2. kinetic perspective. Front Environ Sci 6. 10.3389/fenvs.2018.00101

[74] Pu H, Yuan Y, Qin L, Liu X (2023) pH drives differences in bacterial community β-diversity in hydrologically connected lake sediments. Microorganisms 11:. 10.3390/microorganisms11030676 PMC10056738 36985249

[75] Wang Z, Li J, Zhao P et al (2024) Integrated microbiome and metabolome analyses reveal the effects of low pH on intestinal health and homeostasis of crayfish (*Procambarus clarkii*). Aquat Toxicol 270:106903. 10.1016/j.aquatox.2024.106903 38503037

[76] Cornman RS Jr, Fike JEM J, et al (2018) An experimental comparison of composite and grab sampling of stream water for metagenetic analysis of environmental DNA. PeerJ 6:e5871. 10.7717/peerj.5871 PMC6286662 30568849

[77] Kembel SW, Wu M, Eisen JA, Green JL (2012) Incorporating 16S Gene Copy Number Information Improves Estimates of Microbial Diversity and Abundance. PLOS Comput Biol 8:e1002743. 10.1371/journal.pcbi.1002743 PMC3486904 23133348

[78] Suzuki MT, Giovannoni SJ (1996) Bias caused by template annealing in the amplification of mixtures of 16S rRNA genes by PCR. Appl Environ Microbiol 62:625–630. 10.1128/aem.62.2.625-630.1996 PMC167828 8593063

[79] Větrovský T, Baldrian P (2013) The Variability of the 16S rRNA Gene in Bacterial Genomes and Its Consequences for Bacterial Community Analyses. PLoS ONE 8:e57923. 10.1371/journal.pone.0057923 PMC3583900 23460914

[80] Gloor GB, Macklaim JM, Pawlowsky-Glahn V, Egozcue JJ (2017) Microbiome datasets are compositional: and this is not optional. Front Microbiol 8. 10.3389/fmicb.2017.02224 PMC5695134 29187837

[81] McMurdie PJ, Holmes S (2014) Waste Not, Want Not: Why Rarefying Microbiome Data Is Inadmissible. PLOS Comput Biol 10:e1003531. 10.1371/journal.pcbi.1003531 PMC3974642 24699258

[82] Llewellyn MS, Boutin S, Hoseinifar SH, Derome N (2014) Teleost microbiomes: the state of the art in their characterization, manipulation and importance in aquaculture and fisheries. Front Microbiol 5. 10.3389/fmicb.2014.00207 PMC4040438 24917852

[83] McKenzie VJ, Song SJ, Delsuc F et al (2017) The Effects of Captivity on the Mammalian Gut Microbiome. Integr Comp Biol 57:690–704. 10.1093/icb/icx090 PMC5978021 28985326

