# Supplementary material for: Gut Microbiota and Feeding Patterns of the Antarctic Fairy Shrimp (Branchinecta gaini Daday, 1910): A Metabarcoding Perspective
Source: Microb Ecol. 2026 Jul 2;89:178. doi: 10.1007/s00248-026-02820-4 (PMC13612802; doi:10.1007/s00248-026-02820-4)
Supplement: Supplementary file 1 [file 248_2026_2820_MOESM1_ESM.pdf]

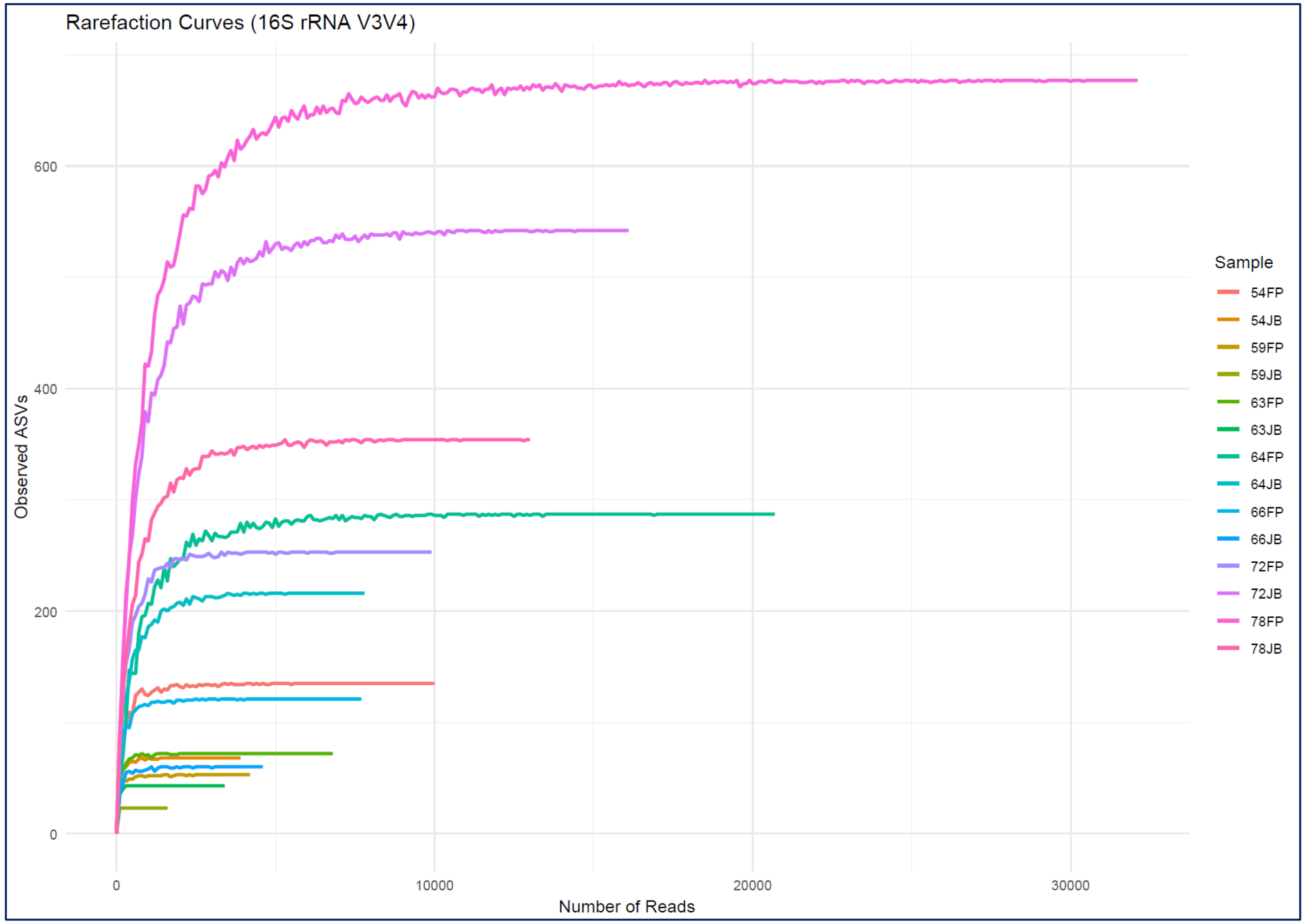

Suplementarny Figure S1. Rarefaction curves of bacterial communities (16S rRNA V3–V4).
Rarefaction curves showing the relationship between sequencing depth (number of reads) and observed amplicon sequence variants (ASVs) for individual samples. Curves approach saturation for most samples, indicating sufficient sequencing depth to capture the majority of bacterial diversity present in the samples.

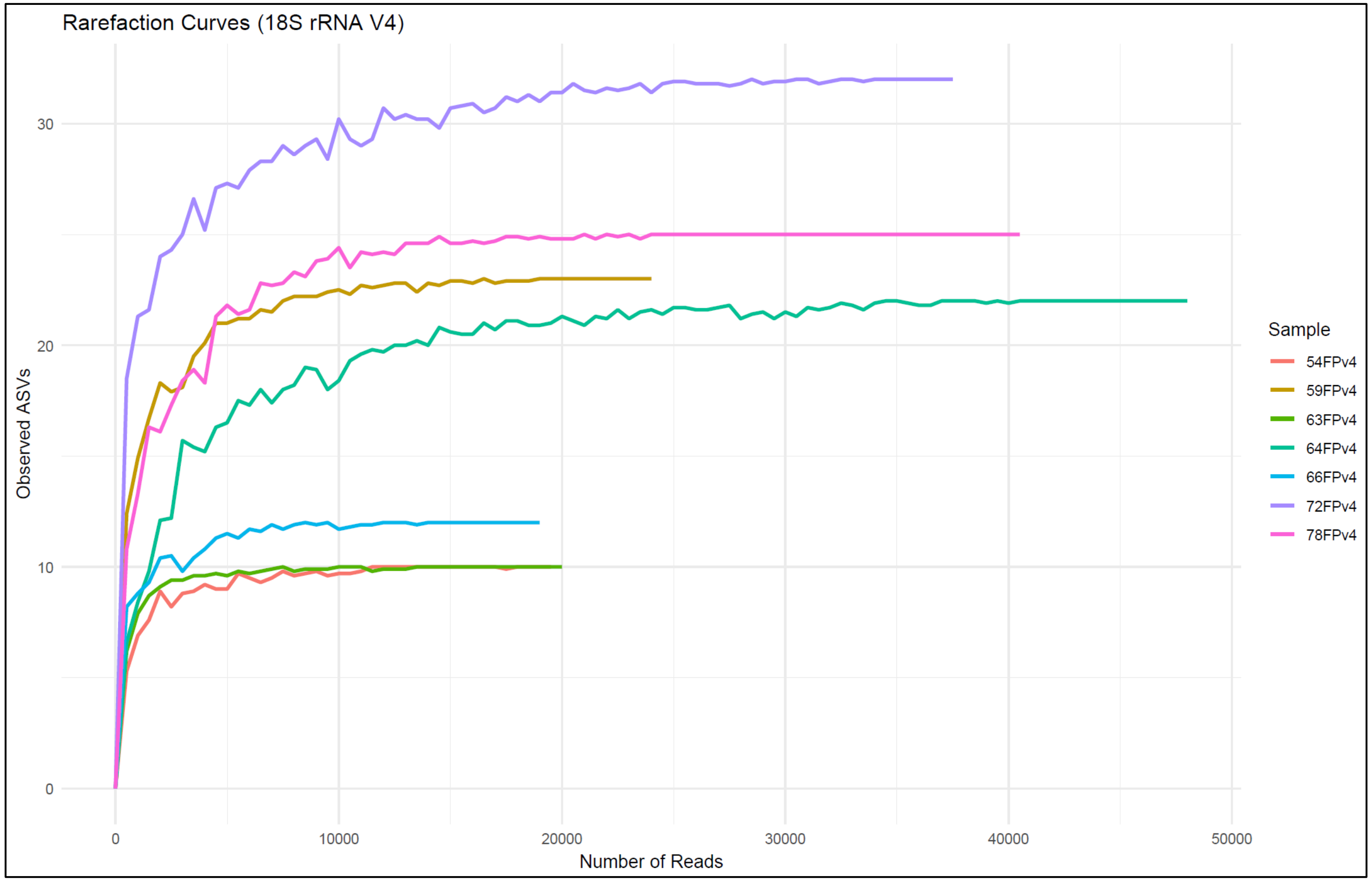

Suplementarny Figure S2. Rarefaction curves of eukaryotic communities (18S rRNA V4).
Rarefaction curves illustrating the accumulation of observed ASVs as a function of sequencing depth for individual samples. Most curves show a tendency toward saturation, suggesting that sequencing depth was sufficient to characterize the dominant eukaryotic diversity, although some samples did not reach a complete plateau.

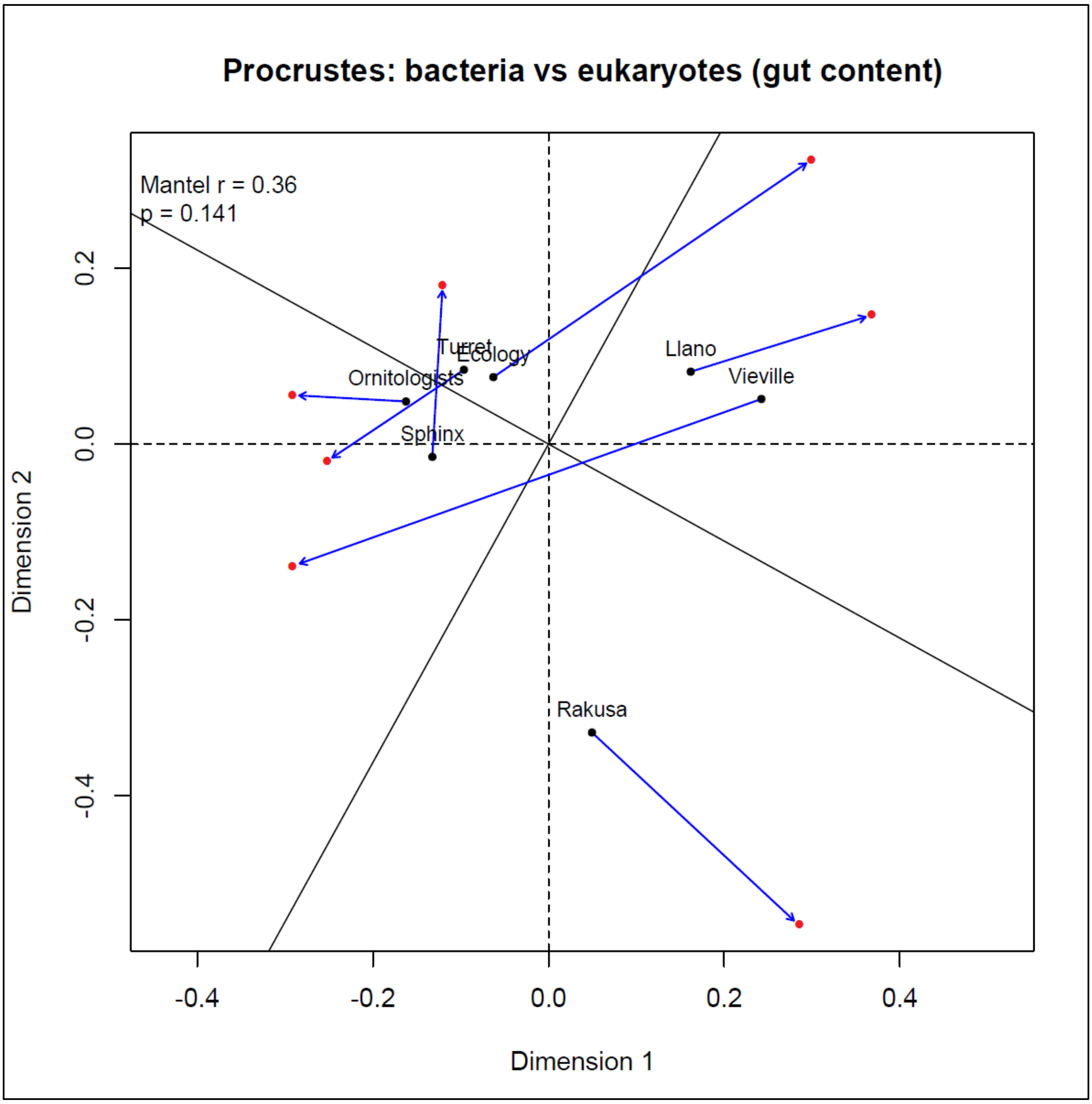

Suplementarny Figure S3. Procrustes analysis comparing bacterial (16S rRNA) and eukaryotic (18S rRNA) community ordinations in gut content samples. The Procrustes plot illustrates the correspondence between bacterial and eukaryotic community structures based on ordinations in reduced-dimensional space. Lines connect matched samples across datasets. The Mantel test indicates a moderate but non-significant correlation between distance matrices ($r = 0.36$, $p = 0.141$).

Supplementary Table S1. Environmental characteristics of sampled ponds

Environmental and spatial characteristics of the eight Antarctic freshwater ponds sampled in this study. For each pond, the table reports its identification number used in the map, pond name, water physicochemical parameters (pH, conductivity, temperature, turbidity), presence or absence of animals, water flow, and bottom characteristics, as well as spatial descriptors including distance to the coast, distance to the nearest glacier, altitude above sea level, and surface area. Binary variables are coded as presence (1) or absence (0). Missing values are indicated as NA.

Number on map	Pond	Water pH	Water conductivity[1]	Water temperature[2]	Water turbidity	Animal presence	Water flow presence	Rocky bottom	Distance to coast[3]	Distance to glacier[3]	Altitude above sea level[3]	Surface area[4]
54	Llano	8.8 ±0.1	461.7 ±3.1	5.6 ±0.1	1	1	0	0	20	520	5	2631.767
59	Ecology	7.8 ±0.1	776.3 ±11.6	4.0 ±0.2	1	1	0	0	43	537	5	6256.875
61	Hennequin	6.7 ±0.1	258.0 ±21.8	NA	0	1	0	0	54	720	5	361.964
63	Viéville	6.4 ±0.2	149.2 ±2.1	3.4 ±0.1	0	1	1	1	44	0	10	47419.57
64	Ornithologists	8.7 ±0.2	246.3 ±115.2	8.2 ±0.2	0	1	1	0	360	512	50	115.271
66	Rakusa	NA	NA	NA	1	1	0	0	42	779	10	12.457
72	Sphinx	7.6 ±0.01	369.8 ±8.9	6.8 ±0.2	1	0	0	0	45	778	5	3139.144
78	Turret	7.5 ±0.02	219.1 ±1.4	7.0 ±0.1	0	1	0	1	238	661	45	1002.141

[1] $\mu S\ cm^{-1}$; [2] °C; [3] m; [4] m^2

Supplementary Table S2. Sequencing read processing statistics for 16S and 18S rRNA gene amplicons. Summary of sequencing read counts at successive stages of amplicon data processing for the bacterial 16S rRNA gene (V3–V4 region) and the eukaryotic 18S rRNA gene (V4 region). For each site and intestinal compartment (gut content and gut tract), the table reports the number of input reads, reads retained after quality filtering, denoising, read merging, chimera removal, and decontamination, along with the percentage of input reads retained at each step. Final read counts used for downstream analyses are reported in the last column. For the 18S rRNA gene, only gut content samples were analyzed.

V3-V4 region of bacterial 16S rRNA gene											
SITE	Compartment	Sample ID	Input	Filtered	Reads retained after filtering (%)	Denoised	Merged	Percentage of input merged	Non-chimeric	Percentage of merged non-chimeric	Reads retained after decontamination (used for analysis)
LLANO	CONTENT	54FP	106613	45115	42.32	42480	29378	27.56	10387	35.3	10064
	TRACT	54JB	82458	34069	41.32	33132	27532	33.39	7356	26.7	3979
ECOLOGY	CONTENT	59FP	56712	24035	42.38	21764	17614	31.06	4444	25.2	4216
	TRACT	59JB	23381	10311	44.1	9552	7069	30.23	2645	37.4	1612
VIÉVILLE	CONTENT	63FP	93315	49169	52.69	47684	42632	45.69	7635	17.9	6849
	TRACT	63JB	40710	18169	44.63	17468	15593	38.3	3964	25.4	3412
ORNITHOLOGISTS	CONTENT	64FP	131127	96704	73.75	94443	90596	69.09	21027	23.2	20764
	TRACT	64JB	104978	67998	64.77	66317	63065	60.07	11064	17.5	7818
RAKUSA	CONTENT	66FP	75378	32510	43.13	27676	16277	21.59	8277	50.8	7763
	TRACT	66JB	62023	27203	43.86	25629	19926	32.13	5352	26.8	4640
SPHINX	CONTENT	72FP	122303	94740	77.46	91664	86825	70.99	14134	16.3	9912
	TRACT	72JB	110777	77465	69.93	74224	66937	60.42	19631	29.3	16138
TURRET	CONTENT	78FP	229938	169223	73.6	161687	146809	63.85	32645	22.2	32173
	TRACT	78JB	102256	74272	72.63	71992	66989	65.51	13934	20.8	13003
V4 region of eukaryotic 18S rRNA gene											
LLANO	CONTENT	54FPv4	26282	20260	77.09	20243	19954	75.92	19721	98.8	1195
ECOLOGY	CONTENT	59FPv4	32977	26366	79.95	26313	25550	77.48	24408	95.5	4746
VIÉVILLE	CONTENT	63FPv4	26593	20868	78.47	20853	20464	76.95	20430	99.8	245
ORNITHOLOGISTS	CONTENT	64FPv4	67786	49482	73	49413	48678	71.81	48397	99.4	473
RAKUSA	CONTENT	66FPv4	30045	19663	65.45	19632	19270	64.14	19251	99.9	468
SPHINX	CONTENT	72FPv4	56633	43660	77.09	43527	42642	75.3	37969	89.1	17421
TURRET	CONTENT	78FPv4	59823	46106	77.07	45944	45642	76.3	40975	89.7	14438

Supplementary Table S3. Results of PERMDISP analyses. Results of permutational analysis of multivariate dispersions (PERMDISP) testing for differences in within-group multivariate dispersion among sample groupings. Tests were based on distances to group centroids calculated from Bray–Curtis dissimilarities using 9,999 permutations. Only groupings yielding stable dispersion estimates are reported.

PERMDISP						
Dataset	Sample grouping	Df	Sums of square	R^2	F-statistics	p-value
ALL	Altitude	3	0.144391	0.04813	59.43	1.118e-06 ***
ALL	Compartment	1	0.0015442	0.0015442	0.7231	**0.4118**
ALL	$Distance^A$	1	0.032117	0.032117	23.21	0.0004207 ***
ALL	$Coast^A$	1	0.048294	0.048294	38.051	4.809e-05 ***
ALL	$Glacier^A$	1	0.000005	0.00000475	0.0017	**0.968**
ALL	$Altitude^A$	1	0.048294	0.048294	38.051	4.809e-05 ***
ALL	$Area^A$	1	0.0030028	0.0030028	1.2285	**0.2894**
ALL	$Distance^M$	1	0.0084786	0.0084786	7.952	0.01546 *
ALL	$Coast^M$	1	0.019050	0.0190495	16.051	0.001742 **
ALL	$Glacier^M$	1	0.000005	0.00000475	0.0017	**0.968**
ALL	$Altitude^M$	1	0.048294	0.048294	38.051	4.809e-05 ***
ALL	$Area^M$	1	0.0030028	0.0030028	1.2285	**0.2894**
$ALL^{\Delta R}$	pH^A	1	0.011510	0.0115100	2.4533	**0.1483**
$ALL^{\Delta R}$	$Conductivity^A$	1	0.0000698	0.00006979	0.0227	**0.8833**
$ALL^{\Delta R}$	$Temperature^A$	1	0.0082059	0.0082059	4.2682	**0.06573**
$ALL^{\Delta R}$	pH^M	1	0.0010882	0.0010882	0.5287	**0.4838**
$ALL^{\Delta R}$	$Conductivity^M$	1	0.013208	0.0132081	6.3308	0.03059 *
$ALL^{\Delta R}$	$Temperature^M$	1	0.0082059	0.0082059	4.2682	**0.06573**

ALL – samples from 7 ponds; $ALL^{\Delta R}$ – samples from 6 ponds (excluding the Rakusa pond); ^{A} – groups defined using the mean value threshold; ^{M} – groups defined using the median value threshold.

Supplementary Table S4. Results of PERMANOVA analyses. Results of permutational multivariate analysis of variance (PERMANOVA) testing for differences in multivariate community composition among sample groupings based on Bray–Curtis dissimilarities using 9,999 permutations. Only factors not significantly affected by heterogeneity of dispersion (PERMDISP) were tested.

PERMANOVA						
Dataset	Sample grouping	Df	Sums of square	R^2	F-statistics	p-value
ALL	Compartment	1	0.3976	0.06937	0.8945	0.779
ALL	$Glacier^A$	1	0.4977	0.08683	1.141	0.141
ALL	$Area^A$	1	0.4447	0.07759	1.0093	0.44
ALL	$Glacier^M$	1	0.4977	0.08683	1.141	0.14
ALL	$Area^M$	1	0.4447	0.07759	1.0093	0.418
$ALL^{\Delta R}$	pH^A	1	0.4711	0.10028	1.1146	0.211
$ALL^{\Delta R}$	$Conductivity^A$	1	0.7344	0.15634	1.8531	**0.003 ****
$ALL^{\Delta R}$	$Temperature^A$	1	0.5119	0.10898	1.223	0.104
$ALL^{\Delta R}$	pH^M	1	0.5400	0.11495	1.2987	0.071
$ALL^{\Delta R}$	$Temperature^M$	1	0.5119	0.10898	1.223	0.105

ALL – samples from 7 ponds; $ALL^{\Delta R}$ – samples from 6 ponds (excluding the Rakusa pond); ^{A} – groups defined using the mean value threshold; ^{M} – groups defined using the median value threshold.
